# PD-1/PD-L blockade in gastrointestinal cancers: lessons learned and the road toward precision immunotherapy

**DOI:** 10.1186/s13045-017-0511-2

**Published:** 2017-08-03

**Authors:** Junyu Long, Jianzhen Lin, Anqiang Wang, Liangcai Wu, Yongchang Zheng, Xiaobo Yang, Xueshuai Wan, Haifeng Xu, Shuguang Chen, Haitao Zhao

**Affiliations:** 1 Department of Liver Surgery, Peking Union Medical College Hospital, Chinese Academy of Medical Sciences and Peking Union Medical College, Beijing, China; 2 Department of General Surgery, Peking Union Medical College Hospital, Chinese Academy of Medical Sciences and Peking Union Medical College, Beijing, China

**Keywords:** Precision immunotherapy, Immune checkpoint blockade, PD-1/PD-L blockade, Gastrointestinal cancer, Biomarker, Combination therapy, Adverse effect, Treatment evaluation, Drug resistance, Cost-effectiveness

## Abstract

Gastrointestinal (GI) malignancies are the most prevalent tumors worldwide, with increasing incidence and mortality. Although surgical resection, chemotherapy, radiotherapy, and molecular targeted therapy have led to significant advances in the treatment of GI cancer patients, overall survival is still low. Therefore, alternative strategies must be identified to improve patient outcomes. In the tumor microenvironment, tumor cells can escape the host immune response through the interaction of PD-1 and PD-L, which inhibits the function of T cells and tumor-infiltrating lymphocytes while increasing the function of immunosuppressive T regulatory cells. The use of an anti-PD-1/PD-L blockade enables reprogramming of the immune system to efficiently identify and kill tumor cells. In recent years, the efficacy of PD-1/PD-L blockade has been demonstrated in many tumors, and this treatment is expected to be a pan-immunotherapy for tumors. Here, we review the signaling pathway underlying the dysregulation of PD-1/PD-L in tumors, summarize the current clinical data for PD-1/PD-L inhibitors in GI malignancies, and discuss road toward precision immunotherapy in relation to PD-1/PD-L blockade. The preliminary data for PD-1/PD-L inhibitors are encouraging, and the precision immunotherapy of PD-1/PD-L inhibitors will be a viable and pivotal clinical strategy for GI cancer therapy.

## Background

Gastrointestinal (GI) cancers are the most common human tumor worldwide, and the incidence and mortality are increasing every year [1, 2]. Several treatment strategies have been developed for GI cancers, including surgery, chemotherapy, radiotherapy, and molecular targeted therapy [3]. These approaches have led to improvements in the treatment of patients with GI cancers. However, the overall survival of GI cancer patients remains poor. Thus, a novel approach to the treatment of GI cancers is needed.

Because the antigens of tumor cells are “self” antigens, the immune system is unable to recognize cancers. Thus, tumors are able to escape the host immune response through a variety of mechanisms at the level of the tumor microenvironment [4]. These mechanisms include but are not limited to (1) the amplification of immunosuppressive cells [e.g., T regulatory cells (Tregs) and myeloid-derived suppressor cells]; (2) the expression of negative co-stimulatory molecules (also known as immune checkpoints) [e.g., cytotoxic T lymphocyte antigen-4 (CTLA-4), programmed death-ligand 1 (PD-1)]; and (3) the secretion of immunosuppressive cytokines and chemokines [e.g., interleukin-10, transforming growth factor-β] [5]. One effective cancer immunotherapy strategy is to use the altered immune system of patients to fight cancer. Early approaches of cancer immunotherapy utilized the transfusion of certain types of cytokines or immune cells, such as high-dose interleukin-2, interferon-α, or cytotoxic T lymphocytes, directly into patients. A considerable number of these studies failed because of the heavy toxicity and low efficacy of the treatments, which was attributed to the probable activation of autoimmune reactions or the immunosuppressive tumor environment [6–8]. Despite these challenges, progress in developing tumor immunology is leading to an era of successful cancer immunotherapy.

Recently, the effectiveness of immunotherapy targeting immune checkpoints in the treatment of numerous forms of cancers has been studied. PD-1, an immune checkpoint, plays a major role in tumor immune escape [9, 10]. The interaction of PD-1 and its ligand PD-L inhibits the function of T cells and tumor-infiltrating lymphocytes (TILs) while increasing the function of immunosuppressive Tregs in the tumor microenvironment [11]. Clinical trials of antibodies against PD-1 and PD-L are being conducted and have demonstrated success in various types of tumors such as advanced melanoma, non-small-cell lung cancer (NSCLC), and renal cell carcinoma (RCC) [12–14]. In this review, we evaluate the current studies and propose precision PD-1/PD-L blockade immunotherapy in GI malignancies including esophageal, stomach, liver, biliary tract, pancreatic, colorectal, and anal cancers.

## PD-1 and its ligands

The myriad of genetic and epigenetic variations and alterations that are features of all cancers supply a varied set of antigens that are utilized by the immune system to distinguish tumor cells from their normal counterparts. Regarding T cells, the ultimate extent and quality of the response is regulated by a balance between co-stimulatory and inhibitory signals, which are initiated through antigen recognition by the T cell receptor (TCR) [15]. Co-stimulatory and inhibitory molecules (also named immune checkpoints) are crucial for the maintenance of self-tolerance and the protection of responses to pathogenic infection under normal physiological conditions. However, the expression of immune checkpoints, an important cancer immune escape and resistance mechanism, can be dysregulated by tumors at both messenger RNA and protein levels [16].

T cells have become the core of cancer immunotherapy efforts owing to their capacities to selectively recognize peptides derived from the cytolysis tumor cells, directly recognize and kill antigen-expressing cells, and integrate adaptive and innate effector mechanisms to orchestrate diverse immune responses such as helper and regulator T cells [17]. Therefore, the blockade of immune checkpoints to reactive T cells mediated antitumor immune responses in a fashion that is transforming human cancer therapeutics.

**Fig. 1:**
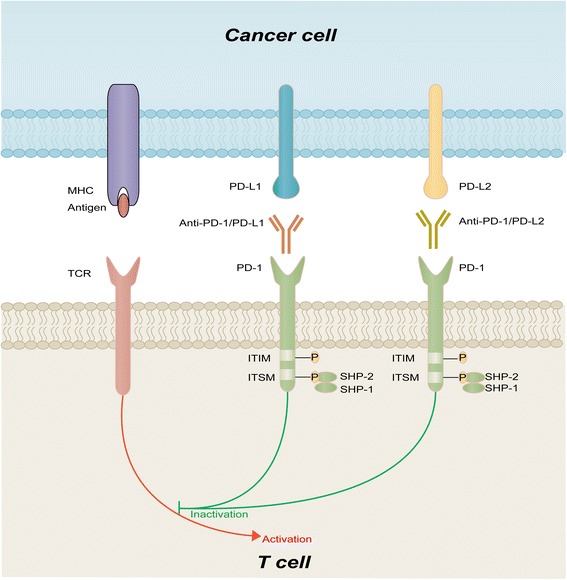
PD-1/PD-L pathway and therapeutic targeting. PD-1 contains an extracellular domain, transmembrane region, and cytoplasmic tail with ITIM and ITSM. During T cell activation through TCR crosslinking with antigen presented by MHC, PD-L1, and PD-L2 expressed on cancer cells downregulate T cell activity by binding to PD-1, unless blocked by anti-PD-1/PD-L1/PD-L2. *Red arrows* indicate inhibitory signals, and *green lines* indicate stimulatory signals

PD-1, also known as CD279, is a cell surface co-inhibitory receptor that induces immune inhibition and promotes tumor immune escape from the cytotoxic T cell immune response during carcinogenesis [18]. PD-1 is predominantly expressed on immunity-associated cells such as T cells, monocytes, B cells and natural killer cells. The PD-1 gene is located on chromosome 2q.37.3 and encodes a type I transmembrane protein belonging to the immunoglobulin superfamily-coordinated stimulus molecule, the main function of which is immunological regulation in autoimmunity, systemic lupus erythematosus, rheumatoid arthritis, viral infection, and transplant immunity as well cancer immunology. The structure of PD-1 is similar to the diverse region of immunoglobulin, and it contains an extracellular domain, a transmembrane region and a cytoplasmic tail. The cytoplasmic tail possesses an immune receptor tyrosine-based inhibitory motif (ITIM) and an immune receptor tyrosine-based switch motif (ITSM) [19]. Studies have demonstrated that the T cell receptor (TCR) signaling pathway can be inhibited by phosphorylation of these two tyrosine motifs (ITIM and ITSM) and further induce the src homology phosphotyrosyl phosphatase (SHP)-1 and SHP-2 proteins, which are essential for the inhibition of T cell activation (Fig. 1).

Several studies have been devoted to the discovery of molecules that interact with PD-1. Programmed cell death ligand-1 (PD-L1), also called B7 homolog 1 (B7-H1) or CD274, was previously identified as an inhibitor of the human T cell response in vitro. PD-L1 was later determined to be a binding and functional partner of PD-1 [20]. Another ligand of PD-1, PD-L2 (B7-DC or CD273), was identified by Latchman et al. in 2001. PD-L2 is selectively expressed on dendritic cells and inhibits TCR-mediated responses through interactions with PD-1 (Fig. 1). Moreover, PD-L1 and PD-L2 do not only interact with PD-1. CD80, a functional ligand for CD28 and CTLA-4, has been shown to collaborate with PD-L1 to mediate an inhibitory signal. Interactions between the PD-1 receptor and its ligands can regulate immune checkpoints, a process that modulates the duration and amplitude of immune responses. These checkpoints are often dysregulated by tumors; thus, they appear to be of extreme importance within a variety of tumors. Moreover, it can lead to the development of an exhausted T cell phenotype characterized by a hierarchical loss of proliferation and cytolytic activity followed by defects in cytokine production and eventually deletion. Therefore, blocking the interactions between PD-1 and its ligands can partly reduce the effect of tumor immune escape and rescue the cytotoxic cell-induced immune response [21].

PD-1 is expressed on a large proportion of tumor-infiltrating lymphocytes (TILs) from many different cancer types. PD-L1 is expressed in 20–50% of human tumors and can provide immune evasion in many cancers by its overexpression (PD-L1 or PD-L2) and an augmented tumor immune response by its (PD-1) abrogated ligand interaction [22]. Based on the crucial role of the PD-1/PD-L pathway in the reciprocal actions between tumor cells and the host immune response, blocking the PD-1/PD-L pathway has been considered a promising and potent therapeutic strategy in tumor inhibition (Fig. 1).

Since tumors can escape the T cell immune response by expressing inhibitory molecules such as PD-1 or PD-L1, blocking the PD-1/PD-L pathway by interfering with binding between PD-1 and its ligands may become a therapy for the treatment of cancer.

## The lessons learned regarding PD-1/PD-L blockade in GI malignancies

### Esophageal cancer

Ranked as the sixth leading cause of cancer-related morbidity worldwide, esophageal cancer is one of the least studied but most lethal medical conditions [23]. Compared with other solid tumors, esophageal cancer has a very high somatic mutation rate [24, 25]. The high mutation load in esophageal tumors has been associated with the clinical benefit of PD-1 blockade [26]. Ohigashi and colleagues evaluated the expression of PD-L1/PD-L2 in 41 esophageal cancer patients and found that 43.9% of samples exhibited PD-L1/PD-L2 overexpression [27].

Pembrolizumab is a PD-1 inhibitor that blocks the interaction between PD-1 and PD-L1 [28]. Doi et al. conducted a phase I clinical trial designed to evaluate the safety and efficacy of pembrolizumab in patients with PD-L1^+^ advanced esophageal carcinoma [28]. PD-L1 expression was assessed using immunohistochemistry (IHC) in tumor or stroma. Of the 23 enrolled patients, the objective response rate (ORR) was 30.4%, and the stable disease (SD) rate was 13.0%. Furthermore, the 6-month and 12-month progression-free survival (PFS) rates were 30.4 and 21.7%, respectively. The authors concluded that pembrolizumab showed meaningful activity in patients with PD-L1^+^ advanced esophageal carcinoma. Similarly, Kojima et al. conducted a phase II study of nivolumab, a fully humanized IgG4 mAb PD-1 inhibitor, in patients with advanced esophageal cancer [29]. Sixty-five patients with esophageal carcinoma who had previously been treated one or more times and were not pre-selected by PD-L1 status were enrolled in this trial, and 64 patients were evaluable for efficacy. The median overall survival (mOS) was 12.1 months, and 17.2% (11 of 64) patients had an objective response (OR). Nivolumab also demonstrated durable antitumor activity in pretreated esophageal cancer. The ORR of pembrolizumab is almost twice that of nivolumab in esophageal cancer, but the trial populations were different: pembrolizumab was used for PD-L1^+^ patients, whereas nivolumab was used for unselected patients. PD-1/PD-L blockade alone or combined with radiotherapy and chemotherapy will be a future research direction in the treatment of advanced esophageal cancer (Table 1).

**Table 1:** The key reported clinical trials of of PD-1/PD-L inhibitors in patients with esophageal cancer

Tumor type	Target	Drug	Phase and identification	Sample size	Clinical end point	TRAEs	Reference
EC	PD-1	Nivolumab	Phase II JapicCTI-142422	64	ORR 17% (central assessment), 22% (investigator assessment); DCR 42% (central assessment), 53% (investigator assessment)	All grade 60%, including diarrhea, decreased appetite, lung infection, rash, fatigue; grade ≥3 17%, including decreased appetite, lung infection, blood creatinine phosphokinase increased, dehydration	Lancet Oncology 2017 [133]
EC	PD-1	Nivolumab	Phase II JapicCTI-142422	64	ORR 17.2%; SD 25.0%; median OS 12.1 m	Drug-related serious AEs 13.8%, including lung infection, dehydration, interstitial lung disease	ASCO 2016 [29]
EC	PD-1	Pembrolizumab	Phase I NCT02054806	23	ORR 30.4%; SD 13.0%; 6-month PFS rate 30.4%; 12-month PFS rate 21.7%	All grade 39.1%, including decreased appetite; grade 3 26%, including decreased lymphocytes	ASCO 2016 [28]
EC	PD-1	Pembrolizumab	Phase Ib NCT02054806	23	ORR 23%; SD 18%; PD 59%	All grade 26%; grade 3 9%	ASCO 2015 [151]

### Gastric cancer

The Cancer Genome Atlas network divides gastric cancer (GC) into four molecular subtypes: (1) Epstein-Barr virus (EBV)-positive tumors, (2) microsatellite instable tumors (MSI), (3) genomically stable (GS) tumors, and (4) tumors with chromosomal instability (CIN) [30]. PD-L1 expression by tumor or tumor-infiltrating immune cells is a general phenomenon in EBV and MSI subtypes, supporting the detection of PD-L1 in these patient populations and the assessment of EBV and MSI status as a key factor in immunotherapy for gastric cancer [31, 32]. Furthermore, the expression of PD-L1 in cancer cells and the microenvironment may contribute to the development of EBV-associated GC, and PD-L1 overexpression is associated with large tumors, lymph node metastasis, and a poor prognosis in gastric cancer [33, 34].

A phase I study of the relationship between PD-L1 expression in advanced gastric cancer patients treated with pembrolizumab and clinical effectiveness was conducted by Muro and colleagues [35]. PD-L1 positivity was determined using a 1% cutoff level for PD-L1 expression in neoplastic cells and contiguous mononuclear inflammatory cells by IHC 22C3 assay. A total of 162 patients were screened for PD-L1 expression, and 65 patients (40%) were PD-L1^+^; a total of 39 patients enrolled in the trial and 36 patients were evaluable for a response. ORR was 33% by investigator review. These results indicated that pembrolizumab exhibited antitumor activity in PD-L1^+^ advanced gastric cancer. Most recently, a clinical phase III trial was conducted to assess the efficacy and safety of nivolumab in patients with unresectable advanced GC/GEC [36]. A total of 493 patients who had failed previous chemotherapy regimens were enrolled. The primary endpoint was OS in the intention-to-treat population. The trial reported that the mOS was 5.32 months among patients with nivolumab versus (vs*.*) 4.14 months among patients with placebo, and the OS rates at 6 and 12 months were 46.4 vs*.* 34.7% and 26.6 vs*.* 10.9%, respectively. The ORR was 11.2% with nivolumab vs*.* 0% with placebo. The median PFS was 1.61 months with nivolumab vs*.* 1.45 months with placebo (Table 2).

**Table 2:** The key reported clinical trials of of PD-1/PD-L inhibitors in patients with gastric cancer

Tumor type	Target	Drug	Phase and identification	Sample size	Clinical end point	TRAEs	Reference
GC/GEC	PD-1	Nivolumab	Phase III NCT02267343	493	ORR 11.2% (nivolumab), 0% (placebo); median PFS 1.61 months (nivolumab), 1.45 months (placebo); median OS 5.32 months (nivolumab), 4.14 months (placebo)	Grade ≥3 11.5% (nivolumab), 5.5% (placebo)	ASCO 2017 [36]
GC/GEC	PD-1	Nivolumab	Phase I/II NCT01928394	59	ORR 12% (all), 18% (PD-L1^+^), 12% (PD-L1^−^); median DOR 7.1 months; median OS 6.8 months; 12-month OS rate 38%	All grade: 66%; grades 3–4 14%, including pneumonitis, fatigue, diarrhea, vomiting, hypothyroidism, increased aspartate and alanine aminotransferase and alkaline phosphatase levels.	ASCO 2016 [78]
GC/GEC	PD-L1	Avelumab	Phase I NCT01772004	75	ORR 15% (2 line group), 7% (switch-maintenance group); median PFS in 2 line group 36.0 weeks (PD-L1^+^), 11.6 weeks (PD-L1^−^); median PFS in switch-maintenance group 17.6 weeks (PD-L1^+^), 11.6 weeks (PD-L1^−^)	TR-TEAEs of any grade 62.7%, including infusion-related reaction; grade ≥3 TR-TEAE 12.0%, including fatigue, thrombocytopenia, and anemia	ASCO 2016 [152]
GC	PD-1	Pembrolizumab	Phase II NCT02335411	259	ORR 11.2% (all), 14.9% (3 line), 7.2% (4 line), 15.5% (PD-L1^+^), 5.5% (PD-L1^−^), 21.3% (3 line with PD-L1^+^), 6.9% (4 line with PD-L1^+^); SD 17%; PD 55.6%; Median DOR: 8.1 months	Grades 3–5 16.6%	ASCO 2017 [153]
GC	PD-1	Pembrolizumab + 5-fluorouracil + cisplatin	Phase II NCT02335411	25	ORR 60% (all), 68.8% (PD-L1^+^), 37.5% (PD-L1^−^); SD 32%; PD 55.6%; median DOR 4.6 months (all), 4.6 months (PD-L1^+^), 5.4 months (PD-L1^−^); median PFS 6.6 months; median OS13.8 months	Grades 3–4 76%	ASCO 2017 [154]
GC	PD-1	Pembrolizumab	Phase I NCT01848834	36	ORR 22% (central review), 33% (investigator review)	Any grade 67%, including fatigue, decreased appetite, hypothyroidism, pruritus and arthralgia; 5 (13%) patients had a total of 6 grades 3–4 TRAEs, including fatigue, pemphigoid, hypothyroidism, peripheral sensory neuropathy, pneumonitis.	Lancet Oncology 2016 [35]
GC	PD-1	Nivolumab; nivolumab + ipilimumab	Phase I/II NCT01928394	154	ORR 16% (all), 14% (nivolumab 3 mg/kg), 26% (nivolumab 1 mg/kg + ipilimumab 3 mg/kg), 10% (nivolumab 3 mg/kg + ipilimumab 1 mg/kg); DCR 38%; 12-month OS rate 36% (nivolumab 3 mg/kg), 34% (nivolumab 1 mg/kg + ipilimumab 3 mg/kg), NA (nivolumab 3 mg/kg + ipilimumab 1 mg/kg); median OS 5.0 months (nivolumab 3 mg/kg), 6.9 months (nivolumab 1 mg/kg + ipilimumab 3 mg/kg), 4.8 months (nivolumab 3 mg/kg + ipilimumab 1 mg/kg)	Any grade 70% (nivolumab 3 mg/kg), 84% (nivolumab 1 mg/kg + ipilimumab 3 mg/kg), 75% (nivolumab 3 mg/kg + ipilimumab 1 mg/kg); grades 3–4: 17% (nivolumab 3 mg/kg), 45% (nivolumab 1 mg/kg + ipilimumab 3 mg/kg), 27% (nivolumab 3 mg/kg + ipilimumab 1 mg/kg)	ASCO 2016 [155]
GC	PD-1	Pembrolizumab	Phase I NCT01848834	39	ORR 22% (central review), 33% (investigator review); median DOR 24 weeks; 6-month PFS rate 24%; 6-month OS rate 69%	4 patients experienced 5 total grades 3–5 TRAEs, including peripheral sensory neuropathy, fatigue, decreased appetite, hypoxia, and pneumonitis; 1 patient experienced drug-related death (hypoxia)	ASCO 2015 [156]
GC	PD-L1	Avelumab	Phase I NCT01943461	11	PR 3 patients	All grades 90.9%, including infusion-related reactions, hyperthyroidism, and pruritus	ASCO 2015 [129]
GC	PD-L1	Durvalumab	Phase I NCT01693562	16	ORR 25%	Any grade (multiple cancer types) 33%, including fatigue, nausea, rash, vomiting, and pyrexia; grade ≥3 (multiple cancer types) 7%	ASCO 2014 [61]
GC	PD-L1	Atezolizumab	Phase I NCT01375842	1	PR 1patient	Grades 3–4 (multiple cancer types) 39%	ASCO 2013 [134]

A number of clinical trials examining PD-1/PD-L blockade combination therapies in advanced gastric cancer have also been performed. The safety and efficacy were investigated for nivolumab as a single agent or in combination with ipilimumab in patients with GC (NCT01928394). Pembrolizumab was evaluated as monotherapy and in combination with cisplatin + 5-fluorouracil in participants with recurrent or metastatic GC/GEC (NCT02335411). Durvalumab monotherapy, durvalumab in combination with tremelimumab, or tremelimumab monotherapy are currently being assessed for the treatment of metastatic or recurrent GC/GEC (NCT02340975).

### Hepatocellular carcinoma and biliary tract cancer

Hepatocellular carcinoma (HCC) is the most common primary liver malignancy [37]. The overall prognosis of HCC patients is poor, and the 5-year survival rate is as low as 12% [38, 39]. A large portion of patients are ineligible for curative resection or transplantation and can only be treated with locoregional therapy or sorafenib, in part because of the late appearance of symptoms [40]. The immune escape pathways of HCC are complex, involving perturbations of antigen presentation and immune effector function, disarray of cytokine profiles, and alterations of immune checkpoint molecules [41–44]. In these mechanisms, PD-1 and PD-L1 play an important role in immune checkpoints. PD-L1 expression ranges from 45 to 100% in HCC samples, and this molecule is highly expressed in tumors and the surrounding antigen-presenting cells [45–48]. Overexpression of PD-L1 is associated with significantly aggressive clinicopathologic features and shorter disease-free survival compared with patients with lower expression levels [47, 49]. Therapeutically, PD-L1 blockade was found to inhibit the growth of HCC tumors in a preclinical xenograft model [50].

Nivolumab was evaluated in a HCC-specific phase I/II study [51]. A total of 262 HCC patients were enrolled. A phase I dose-escalation study evaluated nivolumab (*n* = 48), and a phase II dose-expansion study was initiated in four cohorts (*n* = 214): sorafenib intolerant/naïve, sorafenib progressors, HBV infected and hepatitis C infected. During dose escalation, no maximum tolerated dose was reached. In the dose expansion phase, the ORR was 20% and the 9-month OS rate was 74%. The median duration of response (DOR) was 9.9 months, and the disease control rate (DCR) was 64%. ORRs of 21 and 23% were observed in the uninfected sorafenib-treated and intolerant/naive patients, respectively (Table 3).

**Table 3:** The key reported clinical trials of of PD-1/PD-L inhibitors in patients with hepatocellular carcinoma and biliary tract cancer

Tumor type	Target	Drug	Phase and identification	Sample size	Clinical end point	TRAEs	Reference
HCC	PD-L1	Durvalumab	Phase I/II NCT01693562	39	ORR 10.3%; DCR 33.3%; median OS 13.2 months; 9-month OS rate 62.3%; 12-month OS rate 56.4%	All grades 80.0%, including fatigue, pruritus, elevated AST; Grades 3–4 20.0%, including elevated AST and elevated ALT.	ASCO 2017 [131]
HCC	PD-1	Nivolumab	Phase I/II NCT01658878	262	ORR 23% (sorafenib-naive), 16–19% (sorafenib-experienced); DCR 63% (sorafenib-naive); 12- month OS rate 73% (sorafenib-naive), 60% (sorafenib-experienced)	All grade 77%; Grade ≥3 23.5%, including elevated AST and elevated ALT.	ASCO 2017 [157]
HCC	PD-1	Nivolumab	Phase I/II NCT01658878	262	ORR 20% (dose expansion phase), 23% (sorafenib-naive), 21% sorafenib-treated); median DOR: 9.9 months (dose expansion phase), DCR 64% (dose expansion phase); 9-month OS rate 74% (dose expansion phase)	Grades 3–4 20%	ASCO 2017 [158]
HCC	PD-1	Nivolumab	Phase I/II NCT01658878	48	ORR 15%; median OS 15.1 months; median DOR 23.7 months; 12-month OS rate 59%; 18-month OS rate 48%	All grade 77%, including rash and AST increase; Grades 3–4 20%, including AST increase, lipase and ALT increase	ASCO 2016 [159]
HCC	PD-1	Nivolumab	Phase I/II NCT01658878	39	ORR 23%; CR 5%; PR 18%; 6-month OS rate 72%	Any grade 71%, including AST increase, amylase increase, rash, ALT and lipase increase; grades 3–4 17%, including AST increase, ALT increase and lipase increase	ASCO 2015 [160]
HCC	PD-L1	Durvalumab	Phase I/II NCT01693562	21	12-month DCR 21%	Any grade (multiple cancer types) 33%, including fatigue, nausea, rash, vomiting, and pyrexia; grade ≥3 (multiple cancer types) 7%	ASCO 2014 [61]
BTC	PD-1	Pembrolizumab	Phase Ib NCT02054806	24	ORR 17%; SD 17%; PD 17%	All grade 63%, including pyrexia and nausea; grades 3–4 17%, including anemia, autoimmune hemolytic anemia, colitis, and dermatitis	ECCO 2015 [55]

To further increase the anti-tumor response, it is likewise necessary to disrupt the HCC-associated immune tolerance using combination approaches. Chen et al. reported that sorafenib promoted anti-tumor immunity by reducing PD-1^−^ and Treg^−^-mediated immunosuppression in a mouse model [52]. Nivolumab combined with ipilimumab, another immune checkpoint antibody, is currently being investigated in patients with advanced liver cancer (NCT01658878). Clinical trials of PD-1/PD-L1 blockade combined with molecular targeting are also in progress, such as pembrolizumab plus lenvatinib, a multiple receptor tyrosine kinase inhibitor that works by blocking certain proteins from helping tumor cells divide and grow (NCT03006926), and nivolumab plus galunisertib, a small molecule inhibitor that blocks the transforming growth factor-beta signaling pathway, which plays an important role in epithelial-mesenchymal transition of tumors (NCT02423343).

The expression of PD-L1 was upregulated in intrahepatic cholangiocarcinoma (ICC) tumor tissue and was found to be associated with poor survival, suggesting that PD-1/-L1 inhibitors may serve as adjuvant therapy [53, 54]. In the phase 1 study evaluating pembrolizumab monotherapy for patients with advanced biliary tract cancer (BTC), 24 patients with PD-L1-positive BTC were recruited [55]. The ORR was 17%, and 17% patients had PD. The median DOR was not reached, and the therapy was well tolerated (Table 3).

### Pancreatic cancer

Despite a deep understanding of the genetic mechanisms underlying pancreatic cancer (PC), current therapies for this malignancy are still limited [56]. The immunosuppressive environment surrounding pancreatic tumor appears to be one of major obstacles to the development of successful therapies for this fatal disease [57]. Advances in our understanding of the coordinated activation and immune suppressive mechanisms in PC have led to immunotherapy as a promising approach [58]. In the field of immunocheckpoint inhibitors, CTLA-4 and PD-L1 inhibitors have been studied in PC patients in two clinical trials. A study of ipilumumab, a CTLA-4 inhibitor, in 27 patients with advanced PC was performed [59]. There were no responders, but one patient experienced a delayed response after initial progressive disease. Similarly, no objective response (complete or partial response) was observed in 14 PC patients treated with MDX1105-01, an anti-PD-L1 antibody [60]. Although only a small number of patients received treatment in two trials, the efficacy of immunotherapy for PC has been questioned with such a low response rate. Fortunately, another immunocheckpoint inhibitor, durvalumab, showed activity against PC [61]. The 12-week DCR was 21% (6 of 29 patients), and the ORR was 7% (2 of 29 patients). However, the response rate remains discouraging and may be improved by combination therapy (Table 4).

**Table 4:** The key reported clinical trials of PD-1/PD-L inhibitors in patients with pancreatic cancer

Tumor type	Target	Drug	Phase and identification	Sample size	Clinical end point	TRAEs	Reference
PC	PD-L1	Durvalumab	Phase I/II NCT01693562	29	ORR 7%; 12-week DCR 21%	Any grade (multiple cancer types) 33%, including fatigue, nausea, rash, vomiting, and pyrexia; grade ≥3 (multiple cancer types) 7%	ASCO 2014 [61]
PC	PD-L1	MDX1105-01	Phase I	14	ORR 0%	Grades 3–4 (multiple cancer types) 9%	The New England Journal of Medicine [60]

### Colorectal cancer

The majority of colorectal cancers (CRCs) develop through a CIN pathway, and approximately 15% show defective mismatch repair (dMMR), which can be measured by either the presence of MSI9 or by the lack of DNA mismatch repair proteins [62, 63]. dMMR tumors can have MSI (also called MSI-high) and a somatic mutation frequency more than 10 to 100 times those of proficient MMR (pMMR) tumors [64, 65]. Many studies have shown that dMMR predicts responsiveness to the immune checkpoint blockade [66, 67].

The clinical activity of immune checkpoint blockade with pembrolizuma was evaluated in a phase II study conducted by Le and colleagues [68]. Pembrolizumab was administered to 28 patients with dMMR CRCs and 25 patients with pMMR CRCs. In the dMMR group, the ORR was 50% (14 of 28 patients) and the DCR was 89% (25 of 28 patients). In the pMMR group, 0 of 25 patients (0%) had an objective response, and 4 of 25 patients (16%) had disease control. The median OS was not reached for dMMR and at 6 months for pMMR. For dMMR CRC, the 24-month PFS was 61% and the 24-month OS was 66%. This study suggests that dMMR can be used as a predictor of the clinical benefits of pembrolizumab. However, it is regrettable that patients with pMMR CRCs showed inferior immunotherapy results.

An important phase II study evaluating the clinical activity of nivolumab in patients with dMMR/MSI-H mCRC was reported at the 2017 Gastrointestinal Cancers Symposium of the American Society of Clinical Oncology (ASCO) [69]. Seventy-four patients were treated with nivolumab. The primary endpoint was ORR assessed by the investigator (INV), and the secondary endpoint was ORR assessed by an independent radiology review committee (IRRC). The ORRs were 31% (INV) and 27% (IRRC), and the DCRs were 69% (INV) and 62% (IRRC). The median time to response was approximately 2.7 months (INV/IRRC). Responses were observed in dMMR/MSI-H mCRC patients regardless of the BRAF or KRAS mutation status, tumor PD-L1 expression level and with or without a clinical history of Lynch syndrome.

In these trials, PD-1 inhibitor demonstrated clear efficacy in patients with MSI-H CRC; however, MSS CRC patients still had a low response to PD-1 inhibitor. Fortunately, preclinical studies performed in mice have shown that MEK inhibitors lead to the upregulation of MHC I on tumor cells, inducing T cell infiltration and enhancing PD-L1 activity [70]. Therefore, Bendell and colleagues conducted a clinical trial combining cobimetinib (a MEK inhibitor) and atezolizumab in 23 CRC patients, and the ORR was 17% [70]. Four patients had a partial response, of which three patients were pMMR and one patient was unknown; five patients had SD. The combination of PD-L1 blockade and MEK inhibitors showed a benefit for MSS patients, providing a new immunotherapy method for MSS tumors (Table 5).

**Table 5:** The key reported clinical trials of of PD-1/PD-L inhibitors in patients with colorectal cancer

Tumor type	Target	Drug	Phase and identification	Sample size	Clinical end point	TRAEs	Reference
CRC	PD-1	Pembrolizumab + mFOLFOX6	Phase II NCT02375672	30	ORR 53%; SD 47%; 8-week DCR 100%; median PFS: not reached	Grades 3–4 36.7% (pembrolizumab + mFOLFOX6), 13.2% (pembrolizumab alone)	ASCO 2017 [161]
CRC	PD-1	Pembrolizumab	Electronic medical record	19	ORR 52%; CR 5%; PR 47%; SD 16%; DCR 68%; median OS 16.1 months; 12-month OS rate 79%; median PFS not reached; 12-month PFS rate 54%	Data not available	ASCO 2017 [162]
CRC	PD-1	Nivolumab + ipilimumab	Phase II NCT02060188	27	ORR 41%; SD 52%; DCR (≥12 weeks) 78%; medians for DOR, PFS and OS: not reached	Grades 3–4 37%	ASCO 2017 [163]
CRC	PD-1	Nivolumab	Phase II NCT02060188	74	ORR 31% (INV), 27% (IRRC); DCR 69% (INV), 62% (IRRC); 12-month PFS rate 8.4% (INV), 45.6% (IRRC); median OS not reached; DOR not reached; 6-month OS rate 83.4%; 12-month OS rate 73.8%	Grades 3–4 20%	ASCO 2017 [69]
CRC	PD-1	Pembrolizumab	Phase II NCT01876511	53	ORR 50% (dMMR), 0% (pMMR); DCR 89% (dMMR), 16% (pMMR); median PFS: not reached (dMMR); 2.4 months (pMMR); median OS: not reached (dMMR); 6 months (pMMR)	Data not available	ASCO 2016 [68]
CRC	PD-L1	Atezolizumab + cobimetinib	Phase I NCT01988896	23	ORR 17%	Grades 3–4 34.8%	ASCO 2016 [70]
CRC	PD-1	Nivolumab; Nivolumab + Ipilimumab	Phase II NCT02060188	82	ORR (MSI-H) 27% (nivolumab 3 mg/kg), 15% (nivolumab 3 mg/kg + ipilimumab 1 mg/kg); median PFS (MSI-H) 5.3 months (nivolumab 3 mg/kg), not reached (nivolumab 3 mg/kg + ipilimumab 1 mg/kg); median OS (MSI-H) 16.3 months (nivolumab 3 mg/kg), not reached (nivolumab 3 mg/kg + ipilimumab 1 mg/kg)	Any grade (MSI-H): 79% (nivolumab 3 mg/kg), 85% (nivolumab 3 mg/kg + ipilimumab 1 mg/kg), including diarrhea and fatigue and diarrhea; Grades 3–4 (MSI-H) 21% (nivolumab 3 mg/kg), 31% (nivolumab 3 mg/kg + ipilimumab 1 mg/kg)	ASCO 2016 [67]
CRC	PD-1	Pembrolizumab + radiotherapy/ablation	Phase II NCT02437071	19	interim ORR 9% (pembrolizumab + radiotherapy), 0% (pembrolizumab + ablation)	Any grade 73%, including fatigue, rash, and nausea	ASCO 2016 [164]
CRC	PD-1	Pembrolizumab	Phase II NCT01876511	41	ORR 40% (dMMR CRC), 0% (pMMR CRC), 71% (dMMR other cancers); DCR 90% (dMMR CRC), 11% (pMMR CRC), 71% (dMMR other cancers); median PFS: not reached (dMMR CRC); 2.2 months (pMMR CRC); OS: not reached (dMMR CRC); 5.0 months (pMMR CRC)	Data not available	ASCO 2015 [165]

### Anal cancer

Anal cancer accounts for 2–3% of GI cancers, including squamous cell carcinomas (SCCs), adenocarcinomas, basal cell carcinomas, melanomas and gastrointestinal stromal tumors (GIST) [71]. As the most common malignancy of anal cancer, anal cancer SCC is a rare malignancy associated with infection by human papillomavirus (HPV). Approximately 90% of anal cancers are attributable to HPV infection, and further risk factors for the development of this disease are linked to immune inhibition and autoimmune disorders [72]. Moreover, intratumoral HPV oncoproteins (E6 and E7) upregulate immune checkpoint proteins such as PD-1 to evade immune-mediated cytotoxicity. Therefore, the anti-PD-1 antibody possibly has potent antitumor effectiveness in anal cancer.

NCT02314169 explored the use of the anti-PD-1 antibody nivolumab for the treatment of metastatic SCC of anal cancer [73]. According to the phase 2 results, 37 patients were enrolled and analyzed, all patients received at least one dose of nivolumab and 9 (24%) patients had responses (2 had a complete response and 7 had a partial response). The median PFS was 4.1 months. The 6-month PFS was 38%. The median OS was 11.5 months, and the estimated 1-year OS was 48%. These outcomes indicate that immune checkpoint blockade appears to be a promising approach for patients with SCC of anal cancer. In trial NCT 02314169, all the patients had HPV infection. The high prevalence and association of HPV with anal cancers led to the postulate that the viral interaction of host tumor cells and the surrounding microenvironment could affect immune responses to immune checkpoint inhibitors (Table 6).

**Table 6:** The key reported clinical trials of of PD-1/PD-L inhibitors in patients with anal cancer

Tumor type	Target	Drug	Phase and identification	Sample size	Clinical end point	TRAEs	Reference
AC	PD-1	Pembrolizumab	Phase I NCT02054806	25	ORR (SCCA) 17%; SD (SCCA) 42%; DCR (SCCA) 58%; SD (NSCCA) 1 patient	Any grade 64%, including diarrhea, fatigue, nausea	Annals of Oncology 2017 [166]
SCCA	PD-1	Nivolumab	Phase II NCT02314169	37	ORR 24%	Common AEs: anemia, fatigue, and rash; grade 3 AEs: anemia, fatigue, rash, and hypothyroidism.	Lancet Oncology 2017 [73]
SCCA	PD-1	Nivolumab	Phase II NCT02314169	33	PD 21%; SD 58%; DCR 79%; median PFS 4.1 months	Common AEs: fatigue, nausea, and rash; grade 3: 6 patients, including fatigue pneumonitis, rash, anemia, and hyperglycemia.	ASCO 2016 [130]
SCCA	PD-1	Pembrolizumab	Phase Ib NCT02054806	25	ORR 20%; SD 44%; PD 32%	Any grade 64%, including fatigue, diarrhea and nausea; grades 3–4 8%, including grade 3 general physical health deterioration and grade 3 thyroid-stimulating hormone increased	ECCO 2015 [132]

## The road toward PD-1/PD-L blockade precision therapy

**Fig. 2:**
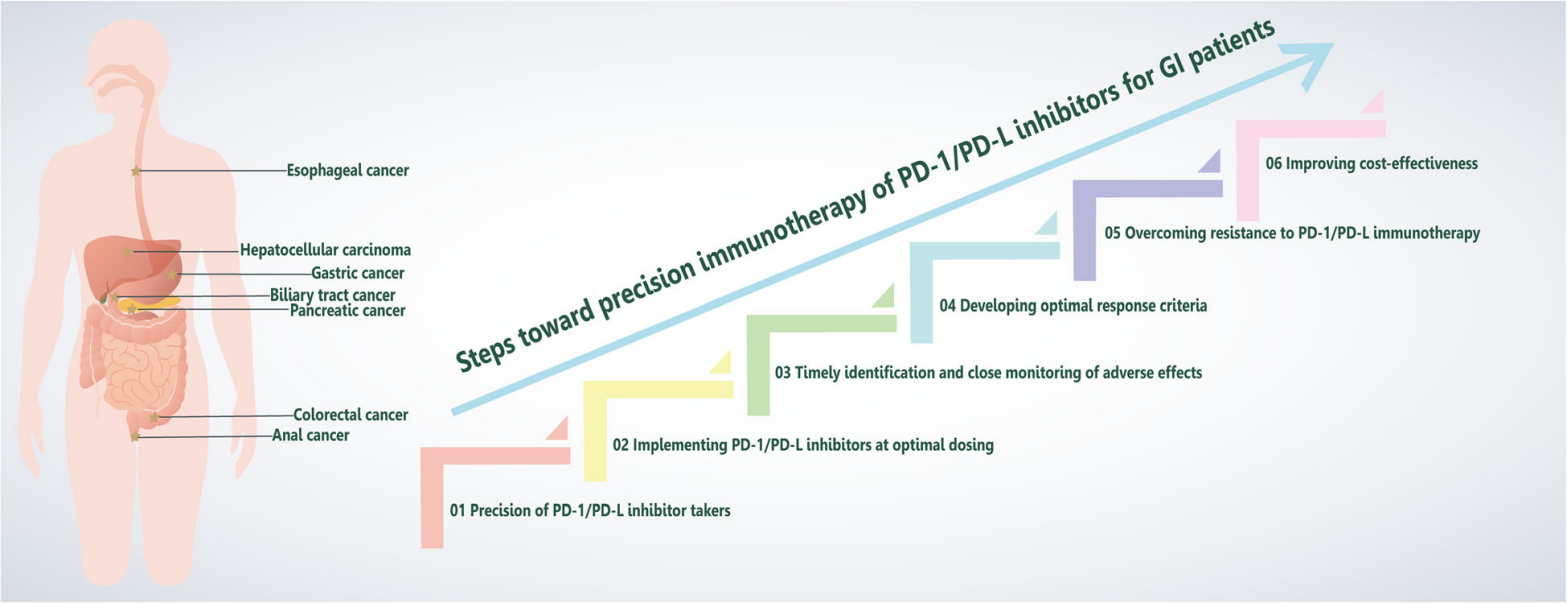
The precision immunotherapy paradigm. GI cancers (*star*) escape the host immune response through the PD-1/PD-L pathway. Although the emergence of PD-1/PD-L blockade has renewed hope in immunotherapy, the response to PD-1/PD-L blockade is not as high as expected. The path toward precision immunology to improve efficiency includes six particularly important steps. The initial step in this process is to identify the population suitable for medication at the time of diagnosis for precision therapy. Once the drug is administered at the optimal time, the patient’s physical condition should be closely monitored, and side effects caused by the drug should be recognized in a timely manner. Concurrently, the efficacy of the drug should be properly evaluated. Upon disease progression, attempts should be made to overcome drug resistance to maintain efficacy. In addition, there is a need to improve the cost-effectiveness ratio to benefit more people. Through these efforts, precision immunotherapy of PD-1/PD-L blockade will become a reality

Precision medicine is broadly defined as “an emerging approach for disease treatment and prevention that takes into account individual variability in genes, environment, and lifestyle for each person” [74]. In the last 5 years, anti-PD-1/PD-L immune checkpoint antibodies have achieved impressive successes in GI cancers [75]. However, a considerable proportion of cancer patients did not respond to PD-1/PD-L, and the drug was not widely available in cancer patients due to its high price. These limitations resulted in challenges for clinical oncologists to develop safer, cheaper and more effective PD-1/PD-L immunotherapies for individual patients, targeting PD-1/PD-L toward precision immunotherapy (Fig. 2).

### Precision of PD-1/PD-L inhibitor consumers

#### Screening of the population suitable for PD-1/PD-L inhibitors

##### PD-L1 and PD-L2 expression

Patients with increased tumor cell and TIL expression of PD-L1 have demonstrated trends toward increased rates of a response to anti-PD1/PD-L1 inhibitors across various clinical trials [76]. However, the detection of PD-L1-negative expression by IHC does not imply a lack of response. Many patients defined as PD-L1-negative using such a biomarker also benefit from PD-1/PD-L inhibitor treatment [77, 78]. Consequently, PD-1/PD-L inhibitors that utilize PD-L1 as an exclusive predictive biomarker are questionable due to many factors [79]. First, the expression of PD-L1 in the tumor has been shown to undergo dynamic changes in different stages of the disease, so the results may be affected by the time of biopsy [76]. Second, there may be considerable heterogeneity in PD-L1 expression within the same cancer as well as between cancer sites, and thus multi-regional sampling is taken into account [79]. Third, PD-L1 expression may not occur simultaneously in immune cells and/or cancer cells [80]. In addition, the cutoff valve of PD-L1 positivity has been defined in different studies, thereby altering the prevalence [81, 82]. Finally, different researchers may use different antibodies and experimental techniques, further affecting interpretations of the results [83, 84]. Based on the above uncertainty, interest has been increasing in the search for alternative biomarkers for responses to immunotherapy. PD-L2 scoring is being evaluated and may provide additional strategies to improve the prediction of PD-1/PD-L inhibitor responses [85]. Yearley and colleagues evaluated the expression of PD-L2 in 172 head and neck squamous cell carcinoma patients treated with pembrolizumab, and they found that PD-L2 positivity was independently associated with longer median durations of OS and PFS [85].

##### Tumor mutation burden

The tumor mutation burden (TMB) is measured by the overall number of somatic protein encoding mutations in the tumor [86]. Tumor cells harboring somatic mutations may produce neoantigens, and the recognition of neoantigens by T cells appears to be crucial for the activity of checkpoint inhibitor immunotherapies [26, 87]. Alexandrov et al. studied the number of mutations in various cancer types and found that lung cancer, melanoma, and bladder cancer with a high mutational load had a high response rate to checkpoint inhibitors [88]. Recently, a study using genomic sequencing investigated the association between TMB and the response to immunotherapy in different solid cancer patients [86]. This study also showed that higher TMB in tumors was associated with a greater likelihood of a response to immunotherapy, regardless of the primary site of cancer. TMB is an informative biomarker in patients who are given an anti-PD-1/PD-L inhibitor. However, it is necessary to explore the best detection methods and cutoff value for the TMB in each tumor. In addition, PD-L1 expression is a relatively mature predictor of the immune response. Techniques to combine TMB and PD-L1 to screen to optimize responses to immunotherapy are also a focus of future studies.

##### MSI/MMR status

The MSI/MMR status can be determined by polymerase chain reaction (PCR) or IHC at specific microsatellite foci [89, 90]. We have demonstrated above that colorectal cancer with dMMR is sensitive to anti-PD-1 antibodies. Additionally, patients with dMMR non-colorectal cancer present responses similar to those of patients with dMMR colorectal cancer [66]. Le et al. investigated the efficacy of PD-1 blockade in patients with advanced dMMR cancers across 12 different tumor types [91]. DCR, ORR and CR were observed in 77, 53, and 21% of patients, respectively. These data suggest that dMMR status has predictive significance for anti-PD-1-directed therapy in all types of cancer patients, regardless of the original tumor location. The Food and Drug Administration (FDA) has granted accelerated approval to pembrolizumab as a treatment for patients with MSI-H or dMMR cancer. This is the first drug based on the biomarker of the tumor, without regard to the cancer tissue origin.

##### Neoantigens, neoantigen intratumoral heterogeneity and MHC antigens

Neoantigens generally established by either somatic mutation genes or viral genes and presented by MHC on the surface of tumor cells have the potential to induce specific anti-tumoral immunity [92]. Next-generation sequencing technology has shown that there are many neoantigens in tumor tissue that may serve as targets for immunotherapies. A study of 110 melanoma patients treated with ipilimumab and analyzed by whole-exome sequencing showed that the neoantigen load served as predictive markers associated with the clinical benefit of ipilimumab [93]. Higher neoantigen burden in tumors was also associated with improved OS, PFS, and durable clinical benefit in NSCLC patients treated with pembrolizumab [26]. In addition, low neoantigen intratumoral heterogeneity may also be important for immune checkpoint inhibitors responses [94]. Melanoma patients with low neoantigen intratumor heterogeneity have shown longer survival times in response to pembrolizumab [94]. In those receiving the neoantigen, the expression of MHC antigens may play a role in the efficacy of immunotherapy [95]. Wang et al. reported that compared with anti-PD1-sensitive tumors, MHC was significantly downregulated in anti-PD1-resistant tumors [96]. Johnson and colleagues showed that the MHC positivity on tumor cells was associated with OS and PFS in a cohort of anti-PD-1-treated melanoma patients [95].

##### Other potential biomarkers and combined biomarkers

Alternative biomarkers, such as tumor etiology, the presence or absence of TILs, composition of TIL effectors, circulating cytokine levels, neutrophil-to-lymphocyte ratio, and baseline and on-treatment immune effector composition, appear to correlate with antitumor activity and represent desirable predictors of responses to immunotherapy [97, 98]. Charoentong et al. revealed genotype–immunophenotype relationships in a pan-cancer immunogenomic analysis and developed a scoring scheme for the quantification, which was termed the immunophenoscore, which predicts the response to PD-1 blockade [99]. Strategies for combining two or more approaches of capturing the immune status of the tumor microenvironment may be more effective as a composite predictive biomarker for the response to anti-PD-1 or anti-PD-L1 monotherapy [94]. Even if the TIL density is low, high expression levels of PD-L1 can be detected in the tumor [100]. Additionally, tumors with high TIL counts may not express PD-L1 [101]. In these two cases, the clinical activity of anti-PD-1/L therapies may be low, but if the expression of PD-L1 or density of TILs alone is used as a biomarker, it may provide an inaccurately high prediction. In a phase I/II trial of 174 advanced HCC patients treated with nivolumab who could be evaluated for PD-L1 expression, objective responses were observed in 19% of 140 patients with PD-L1 <1 and 26% of 34 patients with PD-L1 ≥1% [51].There was no significant difference in the OS rate between groups based on the expression of PD-L1. One possible reason for these findings is that only the expression of PD-L1 was considered in the trial, without considering the number of infiltrating TILs. If only PD-L1 is overexpressed without infiltration of cytotoxic T lymphocytes, immune checkpoint inhibitors are naturally ineffective toward such “cold tumors.” The combination of CD8 protein and PD-L1 expression may predict patients who will respond to nivolumab. Therefore, the combination of biomarkers is a potential research direction for clinical trials. The future development of an effective biomarker for predicting responses to anti-PD-1 or anti-PD-L1-based therapies will integrate multiple methods for optimal characterization of the immune tumor microenvironment.

#### Turning patients with no response to PD-1/PD-L inhibitor into those with a response to PD-1/PD-L inhibitor and improving efficacy

Immunotherapy can provide patients with a better clinical effect, and we also note that unselected patients who receive anti-PD-1 and anti-PD-L1 immunotherapy have a response rate of only approximately 20%, necessitating other treatment strategies to allow the remaining 80% non-responders to be converted to responders. Radiation therapy has the advantage of interfering with the primary tumor site and potentially restoring some of the established immunosuppressive barriers present in the tumor microenvironment, ideally restoring the primary tumor as an effective immunogenic center. Local radiation also triggers a systemic effect that can be used in combination with immunotherapy to elicit a response external to the radiation field [102]. Two trials have examined the combination of nivolumab with radiation therapy in glioblastoma (NCT02617589) and advanced non-small cell lung cancer (NCT02768558). High levels of vascular endothelial growth factor (VEGF) hinder dendritic cell functions, and VEGF-targeted therapy enhances immune checkpoint molecule expression by reducing VEGF levels, suggesting that the combination of PD-1/PD-L and VEGF antibody merit further study [103]. A clinical trial using a combination of bevacizumab (anti-VEGF antibody) and ipilimumab has reported beneficial initial results in melanoma patients [104]. Dual immune checkpoint blockade by combining anti-PD-1 and anti-CTLA-4 treatment also enhances anti-tumor effects by targeting different activation mechanisms of T cells. In a study of patients with advanced melanoma, treatment with a combination of nivolumab and ipilimumab or nivolumab alone resulted in significantly longer PFS and higher ORR than ipilimumab alone [105]. Therefore, the development of strategies for future treatments combining PD-1/PD-L blockade with chemotherapy, radiotherapy, targeted agents and or other immunotherapy agents, especially for cancer patients with negative or weakly positive PD-L1 expression, will be taken into account. However, with the increase in response rates, a greater number of immune-related adverse events have also been observed, and many patients have been unable to complete the combination therapy in clinical trials, resulting in concerns about a trade-off between drug tolerance and efficacy in combination therapy.

#### Possible unsuitability of PD-1/PD-L inhibitor users

Although anti-PD-1/L monotherapy can lead to profound and sustained tumor responses in some cases, a small subset of patients treated with anti-PD-1/L inhibitor appear to exhibit hyperprogression of disease (HPD) [106]. Compared with before treatment, the tumor growth rate (TGA) and clinical deterioration of these patients were greatly accelerated [107]. Champiat and colleagues defined HPD as a ≥2-fold increase in the tumor growth rate in patients with disease progression and estimated that at least 9% of cases overall are likely to present HPD [106]. Thus, it is imperative to identify predictors of HPD, some of which include the following: (1) HPD significantly correlates with older age, especially an age ≥65 years [106], which may be due to the different immune background of elderly patients [108, 109]. Patients older than 65 years should be pay greater attention when using anti-PD-1/PD-L1. (2) HPD is associated with regional recurrence [110]. Prior irradiation may play a key role since almost all cases of hyperprogression occur in patients with at least locoregional recurrence at the site of irradiation [110]. (3) Some patients with MDM2/4 amplification or EGFR aberrations have shown remarkably accelerated TGA after anti-PD1/PD-L therapy, indicating the need for caution in the presence of these genomic spectra [107]. Hyperprogression may result from the ability of MDM2 amplification to inhibit the p53 tumor suppressor [111]. Individuals with these risk factors treated with anti-PD1/PDL1 monotherapy should be closely monitored.

### Optimal timing of implementing a PD-1/PD-L inhibitor

Although combination therapy is becoming more prevalent, few studies are designed to optimize clinical efficacy based on the timing of administration. In fact, timing is another critical factor for determining the outcome of immunotherapy, and the optimal timing varies [112]. Radiation therapy of tumors modulates the peptide repertoire, resulting in a dose-related increase in MHC class I expression [113, 114]. Maximum loading of the tumor stroma with tumor antigen occurred 2 days following high-dose radiation [115]. Many pre-clinical and clinical immunotherapies targeting T cells therefore are applied closely following radiation [116, 117]. These approaches have been shown to increase the tumor-antigen specific immune response to varying degrees. A retrospective study evaluated the OS of metastatic lung cancer patients who received radiotherapy within 30 days preceding (Before) or during (Sandwich) nivolumab treatment [118]. Among 76 metastatic lung cancer patients treated with nivolumab, 22 received radiotherapy—10 Before and 12 Sandwich. The median OS for patients with no radiotherapy was 4.8 months; Before was 5.2 months and Sandwich was not reached. An improvement in OS was observed when radiotherapy was administered as a Sandwich approach during nivolumab treatment. Alterations in the drug design also resulted in different immunogenic properties. Chemotherapeutic drugs may modulate the tumor and its microenvironment to potentiate anti-tumor immune responses [119]. A phase II study of advanced lung cancer assessed the activity of ipilimumab in combination with chemotherapy [120]. In that study, 204 patients were randomly assigned 1:1:1 to receive chemotherapy alone or four doses of ipilimumab plus chemotherapy followed by two doses of placebo plus chemotherapy or two doses of placebo plus chemotherapy followed by four doses of ipilimumab plus chemotherapy. The median OS was 8.3, 9.7, and 12.2 months, respectively, which suggested that chemotherapy followed by immunotherapy plus chemotherapy may achieve better efficacy. Many targeted therapies can modulate T cell proliferation and the immune response to tumor antigens [121]. The mTOR pathway has been well characterized in the modulation of cell growth and metabolism [122]. When administered prior to immunostimulation, mTOR inhibitor may amplify the population of regulatory T cells, whereas continuous mTOR inhibition after immunostimulation may hinder both regulatory T cells and effector T cells equally, indicating that the optimal timing of particular combinations of targeted agents and immunotherapy must also be precisely investigated to maximize anti-tumor effects [122, 123]. However, additional data are needed to guide clinical practice. In addition, cancer-specific immunotherapy may be greater with a lower tumor burden [124–126]. Therefore, cancer patients should receive PD-1/PD-L inhibitor treatment in the early stage rather than the advanced stage of tumor development.

### Timely identification and close monitoring of adverse effects

Immunotherapy can result in a unique spectrum of immune-related adverse effects (irAEs) [76]. However, anti-PD-1 or anti-PD-L1 antibodies are well tolerated at a wide range of therapeutic doses compared with molecular-targeted agents and cytotoxic anticancer agents, which also damage the quality of life of patients [127]. In patients with GI cancers, irAEs of any grade appear in 26–90.9% of patients [36, 128, 129]. Common irAEs include anemia, vomiting, nausea, pyrexia, fatigue, rash, colitis, increased thyroid-stimulating hormone, and elevated aspartate aminotransferase (AST) and alanine aminotransferase (ALT) [55, 61, 73, 130–132]. It is noteworthy that predictable irAE patterns were observed in such patients; early onset of dermatological and gastrointestinal toxicity, late emergence of liver toxicity or endocrine disorders. Many of these adverse events can be controlled by withdrawing the PD-1 and PD-L1 inhibitors and initiating steroid therapy. Additionally, grades 3–4 irAEs including autoimmune hemolytic anemia, hepatitis, inflammatory colitis and pneumonitis were observed in 7–39% of patients with GI tumors receiving single PD-1/PD-L1 blockade [61, 133, 134]. Colitis and pneumonitis are monitored very closely in all patients on PD-1 blockade. When combined with other agents (especially other immunotherapeutic agents), the incidence and severity of these adverse events are amplified [67]. If serious grades 3–4 toxicity occurs, intravenous steroids should be administered, and the checkpoint inhibitors should be discontinued permanently. In addition, it is well established that the incidence of irAE with PD-1/PD-L1 inhibitors is underestimated in clinical trials. Patients in the real world may be frailer with more complications than patients in clinical trials, indicating that entire irAEs are expected to be much higher in the real world. It is expected that ongoing trials will further reduce risk and improve the clinical efficacy of PD-1 and PD-L1 inhibitors by raising awareness, identifying, and managing these risks over time.

### Developing optimal response criteria

There are several criteria for assessing tumors, including the World Health Organization (WHO), modified WHO, RECIST 1.0, RECIST 1.1, and modified RECIST criteria. RECIST and mWHO criteria are used in clinical trials to assess responses to cytotoxic chemotherapy [38, 135]. Unlike responses observed using conventional cytotoxic chemotherapy, immunotherapy is associated with alternative clinical response patterns. In some cases, a small percentage of patients exhibit early visible progression of the disease by RECIST criteria before a long-term immune-related clinical response. Because of inflammatory cell infiltration and/or necrosis, pseudoprogression occurs after PD-1/PD-L blockade in several solid tumors, and an improved outcome is apparent in these patients [98, 136]. Moreover, PD-1/PD-L blockade affects the host anti-tumor response, which requires some time to achieve a measurable or sustained clinical efficacy compared to conventional cytotoxic chemotherapy. Consequently, immune-related response criteria (irRC) were developed to evaluate the efficacy of PD-1/PD-L blockade [137]. All lesions are considered with the total tumor burden evaluated at each scan rather than a defined target lesion using irRC criteria. Suspected disease progression in asymptomatic patients in one scan requires confirmation of the scan in approximately four to six weeks, during which time the patient can remain on treatment [97]. IrRC can more accurately assess the response to anti-PD-1/L therapy compared with RECIST or WHO criteria [138]. However, irRC is also facing many challenges. For example, tumor burden is the sum of all the target lesions, which accounts for high interobserver variability, and measuring tumor burden is time consuming [139]. Future perspective studies are needed to determine the consensus on optimal radiological criteria or the combination of criteria for patients with PD-1/PD-L blockade.

### Strategies after resistance to PD-1/PD-L immunotherapy

#### Combined specific targeting drugs

Despite the compelling anti-tumor efficacy of antibodies targeting the PD-1/PD-L immune checkpoint in a variety of cancers, many patients do not respond to therapy, and more concerning, the initial response of some patients to immunotherapy showing encouraging results eventually leads to drug resistance. A recent study showed that of 78 patients with melanoma treated with a PD-1 inhibitor, 42 had an objective response and 15 subsequently developed disease progression [140]. The researchers analyzed and compared the whole genome sequence of tumor cells in four patients before and after treatment with the PD-1 inhibitor. One of the patient’s tumor cells lost a gene called B2M, which alters the way the immune system recognizes cancer cells. Tumors from two other patients had a JAK gene mutation, limiting the ability of the immune system to kill cancer cells. These observations confirm that tumors can be resistant to PD-1 inhibitor by gene mutations [140, 141]. Another study revealed increased expression of TIM3 in TILs after anti-PD-1 treatment in a mouse model, and the combination of anti-PD-1 and TIM3 inhibitors significantly inhibited tumor growth and prolonged mouse survival [142]. Therefore, as PD-1/PD-L pathway resistance mechanisms are being elucidated, effective treatment patterns will be established.

#### Continued use of the PD-1/PD-L immune checkpoint

Improved survival and tumor reduction after RECIST-defined progression was observed in a subset of patients [143]. Immunotherapy can have a positive effect on the PFS effect or OS response rate due to tumor immune infiltration or delayed response [144]. A phase III study of atezolizumab evaluated post-PD OS and safety in patients with non-small cell lung cancer [144], in which of 168 patients with PD who continued atezolizumab treatment beyond RECIST progression (TBP), 7% achieved a subsequent response in target lesions and 49% had stable target lesions. Similarly, a subgroup analysis of patients treated with nivolumab beyond RECIST-defined progression was conducted in a phase 3 study [143]. Among the 153 patients with advanced RCC TBP with nivolumab, 142 patients with pre-progression and post-progression tumor measurements were evaluable. Of all patients, 13% experienced a subsequent ≥30% reduction in tumor burden. It is noteworthy that TBP was allowed if patients tolerated therapy and showed the clinical benefit of the investigator’s assessment. PD-1 treatment may be continued in previously treated patients with good physical condition. However, further research is necessary to better identify the patients who may benefit from TBP.

#### Chemotherapy after resistance to PD-1/PD-L inhibitor

Immune checkpoint inhibitors are active for advanced cancer patients who have progressed following chemotherapy [145]. A retrospective case–control study was conducted to determine whether salvage chemotherapy could provide additional benefit to patients who have not responded to immune checkpoint inhibitors or progressed after initial response to these agents [146]. Among 82 patients with advanced NSCLC, 67 patients had received a PD-1/PD-L1 inhibitor (case group) and 15 patients had received prior chemotherapy or chemoradiotherapy only (control group). Eighteen case group patients and only 1 control group patient experienced PR with salvage chemotherapy. The odds ratio for achieving PR was 0.30 (27 vs*.* 7%), and no significant differences in the likelihood of obtaining PR were found according to sex, age, tumor histology, type of salvage chemotherapy regimen and number of prior chemotherapy regimens, indicating that patients with advanced NSCLC who have progressed following treatment with a PD-1/PD-L1 checkpoint inhibitor have a 30% better chance of achieving at least PR with salvage chemotherapy compared with those who have received prior chemotherapy but not a PD-1/PD-L1 checkpoint inhibitor. Immunotherapy can alter the natural history and microenvironment of the tumor, making it more sensitive to chemotherapy. These preliminary findings may facilitate the development of a new approach to drug resistance to immunotherapy.

### Improving PD-1/PD-L inhibitor cost-effectiveness

Despite advances across various tumors, it is recommended that the high cost of PD-1/PD-L1 inhibitors be carefully evaluated to ensure their economic sustainability for the health care industry and benefit to all cancer patients [147]. In this regard, assessments of quality-adjusted life year (QALY) and incremental cost-effectiveness ratios (ICERs), as well as the impact of drug reimbursement patterns, are the main focuses of pharmaceutical economists [147]. According to the current cost of nivolumab for metastatic RCC patients in the USA, the ICER for nivolumab vs*.* everolimus ($151,676/QALY) is beyond the willingness-to-pay (WTP) threshold of $100,000/QALY [148]. The chance of nivolumab being cost-effective is low [149]. However, nivolumab should not be overlooked due to its superior tolerability and benefit to everolimus [149]. The ICER is very sensitive to the price of nivolumab [149]. A cost decrease of nivolumab by 13% would take the ICER below the WTP threshold [149]. If the cost is reduced by 40%, the chance of nivolumab being cost-effective would be as high as 100%; this suggests that a price reduction seems reasonable [149]. In addition, nivolumab is not cost-effective compared with treatment with docetaxel for non-squamous NSCLC at the current cost in Switzerland [150]. However, the cost-effectiveness of nivolumab improves by reducing the dose, treatment duration or drug price and selecting PD-L1-positivite patients [150]. Although the cost-effectiveness analysis of the PD-1/PD-L1 inhibitor for GI tumor patients has not yet been reported, it is foreseeable that it will be improved by developing alternative agents, reducing drug costs and selecting appropriate patients.

## Conclusion

The clinical data from GI tumor trials has demonstrated that immunotherapy targeting immunocheckpoints have produced exciting clinical benefits. However, the response rate is not as high as expected, and therefore treatment with PD-1/PD-L inhibitors must be subjected to precision immunotherapy to improve efficiency. Ongoing and future research should explore the genetic and molecular mechanisms involved in the response and resistance to PD-1/PD-L inhibitors and develop a correct criterion for evaluating the efficacy of PD-1/PD-L blockade. It will also be important to identify predictable and reliable combined biomarkers that will help to select patients who may benefit from PD-1/PD-L inhibitors while minimizing toxicities and maximizing cost-effectiveness. After integrating these approaches, individualized and precise immunotherapies will hopefully lead to a more effective treatment, perhaps even conquest, of GI tumors.

## Abbreviations

AC: Anal canal
AEs: Adverse events
ASCO: American Society of Clinical Oncology
B2M: Beta-2-microglobulin
B7-H1: B7 homolog1
BRAF: B-Raf proto-oncogene
BTC: Biliary tract cancer
CIN: Chromosomal instability
CRC: Colorectal cancer
CTLA4: Cytotoxic T-lymphocyte antigen-4
DCR: Disease control rate
dMMR: Mismatch repair deficient
DOR: Duration of response
EBV: Epstein-Barr virus
EC: Esophagus cancer
ECCO: European Cancer Congress
GC: Gastric cancer
GEC: Gastroesophageal junction cancer
GI: Gastrointestinal
GIST: Gastrointestinal stromal tumors
GS: Genomically stable
HCC: Hepatocellular carcinoma
HPV: Human papillomavirus
INV: Investigator
irRC: Immune-related response criteria
IRRC: Independent radiology review committee
JAK: Janus kinase
KRAS: KRAS proto-oncogene
MEK: MAP kinse-ERK kinase
MHC: Major histocompatibility complex
mOS: Median overall survival
MSI-H: Microsatellite instability-high
MSS: Microsatellite stability
NSCCA: Non-squamous cell carcinoma of the anal canal
ORR: Objective response rate
OS: Overall survival
PC: Pancreatic cancer
PD: Progressive disease
PD-1: Programmed cell death-1
PD-L1: Programmed cell death ligand-1
PD-L2: Programmed cell death ligand-2
PFS: Progression-free survival
pMMR: Mismatch repair proficient
PR: Partial response
RECIST: Response evaluation criteria in solid tumors
SCCA: Squamous cell carcinoma of the anal canal
SCCs: Squamous cell carcinomas
SD: Stable disease
SHP-1: Src homology phosphotyrosyl phosphatase-1
SHP-2: Src homology phosphotyrosyl phosphatase-2
TCR: T cell receptor
TILs: Tumor-infiltrating lymphocytes
TRAEs: Treatment-related adverse events
Tregs: T regulatory cells
TR-TEAEs: Treatment-related treatment-emergent adverse events
VEGF: Vascular endothelial growth factor
WHO: World Health Organization

## References

[1] Ferlay J, Soerjomataram I, Dikshit R, Eser S, Mathers C, Rebelo M, Parkin DM, Forman D, Bray F (2015). Cancer incidence and mortality worldwide: sources, methods and major patterns in GLOBOCAN 2012. Int J Cancer.

[2] Ahmad A, Reha J, Abdul S, Joseph Espat N, Somasundar P, Katz SC (2017). Association of primary tumor lymph node ratio with burden of liver metastases and survival in stage IV colorectal cancer. HepatoBiliary Surgery and Nutrition.

[3] Kim JH, Kim BJ, Kim HS, Kim JH (2016). Current Status and Perspective of Immunotherapy in Gastrointestinal Cancers. J Cancer.

[4] Kroemer G, Galluzzi L (2015). Combinatorial immunotherapy with checkpoint blockers solves the problem of metastatic melanoma-An exclamation sign with a question mark. Oncoimmunology.

[5] Schreiber RD, Old LJ, Smyth MJ (2011). Cancer immunoediting: integrating immunity’s roles in cancer suppression and promotion. Science.

[6] Parkinson DR, Abrams JS, Wiernik PH, Rayner AA, Margolin KA, Van Echo DA, Sznol M, Dutcher JP, Aronson FR, Doroshow JH (1990). Interleukin-2 therapy in patients with metastatic malignant melanoma: a phase II study. J Clin Oncol.

[7] Flaherty LE, Atkins M, Sosman J, Weiss G, Clark JI, Margolin K, Dutcher J, Gordon MS, Lotze M, Mier J (2001). Outpatient biochemotherapy with interleukin-2 and interferon alfa-2b in patients with metastatic malignant melanoma: results of two phase II cytokine working group trials. J Clin Oncol.

[8] Slankard-Chahinian M, Holland JF, Gordon RE, Becker J, Ohnuma T (1984). Adoptive autoimmunotherapy. Cytotoxic effect of an autologous long-term T-cell line on malignant melanoma. Cancer.

[9] Okazaki T, Honjo T (2006). The PD-1-PD-L pathway in immunological tolerance. Trends Immunol.

[10] Keir ME, Liang SC, Guleria I, Latchman YE, Qipo A, Albacker LA, Koulmanda M, Freeman GJ, Sayegh MH, Sharpe AH (2006). Tissue expression of PD-L1 mediates peripheral T cell tolerance. J Exp Med.

[11] Dong Y, Sun Q, Zhang X (2017). PD-1 and its ligands are important immune checkpoints in cancer. Oncotarget.

[12] Robert C, Schachter J, Long GV, Arance A, Grob JJ, Mortier L, Daud A, Carlino MS, McNeil C, Lotem M (2015). Pembrolizumab versus Ipilimumab in Advanced Melanoma. N Engl J Med.

[13] Garon EB, Rizvi NA, Hui R, Leighl N, Balmanoukian AS, Eder JP, Patnaik A, Aggarwal C, Gubens M, Horn L (2015). Pembrolizumab for the treatment of non-small-cell lung cancer. N Engl J Med.

[14] Motzer RJ, Escudier B, McDermott DF, George S, Hammers HJ, Srinivas S, Tykodi SS, Sosman JA, Procopio G, Plimack ER (2015). Nivolumab versus Everolimus in Advanced Renal-Cell Carcinoma. N Engl J Med.

[15] Gratz IK, Rosenblum MD, Maurano MM, Paw JS, Truong HA, Marshak-Rothstein A, Abbas AK (2014). Cutting edge: Self-antigen controls the balance between effector and regulatory T cells in peripheral tissues. J Immunol.

[16] Kean LS, Turka LA, Blazar BR (2017). Advances in targeting co-inhibitory and co-stimulatory pathways in transplantation settings: the Yin to the Yang of cancer immunotherapy. Immunol Rev.

[17] Boussiotis VA (2016). Molecular and Biochemical Aspects of the PD-1 Checkpoint Pathway. N Engl J Med.

[18] Dai S, Jia R, Zhang X, Fang Q, Huang L (2014). The PD-1/PD-Ls pathway and autoimmune diseases. Cell Immunol.

[19] Zak KM, Kitel R, Przetocka S, Golik P, Guzik K, Musielak B, Domling A, Dubin G, Holak TA (2015). Structure of the Complex of Human Programmed Death 1, PD-1, and Its Ligand PD-L1. Structure.

[20] Yao S, Zhu Y, Chen L (2013). Advances in targeting cell surface signalling molecules for immune modulation. Nat Rev Drug Discov.

[21] Villalba M, Rathore MG, Lopez-Royuela N, Krzywinska E, Garaude J, Allende-Vega N (2013). From tumor cell metabolism to tumor immune escape. Int J Biochem Cell Biol.

[22] Zou W, Wolchok JD, Chen L (2016). PD-L1 (B7-H1) and PD-1 pathway blockade for cancer therapy: Mechanisms, response biomarkers, and combinations. Sci Transl Med.

[23] Pennathur A, Gibson MK, Jobe BA, Luketich JD (2013). Oesophageal carcinoma. Lancet.

[24] Segal NH, Parsons DW, Peggs KS, Velculescu V, Kinzler KW, Vogelstein B, Allison JP (2008). Epitope landscape in breast and colorectal cancer. Cancer Res.

[25] Lawrence MS, Stojanov P, Polak P, Kryukov GV, Cibulskis K, Sivachenko A, Carter SL, Stewart C, Mermel CH, Roberts SA (2013). Mutational heterogeneity in cancer and the search for new cancer-associated genes. Nature.

[26] Rizvi NA, Hellmann MD, Snyder A, Kvistborg P, Makarov V, Havel JJ, Lee W, Yuan J, Wong P, Ho TS (2015). Cancer immunology. Mutational landscape determines sensitivity to PD-1 blockade in non-small cell lung cancer. Science.

[27] Ohigashi Y, Sho M, Yamada Y, Tsurui Y, Hamada K, Ikeda N, Mizuno T, Yoriki R, Kashizuka H, Yane K (2005). Clinical significance of programmed death-1 ligand-1 and programmed death-1 ligand-2 expression in human esophageal cancer. Clin Cancer Res.

[28] Doi T, Piha-Paul SA, Jalal SI, Mai-Dang H, Saraf S, Koshiji M, Csiki I, Bennouna J (2016). Updated results for the advanced esophageal carcinoma cohort of the phase Ib KEYNOTE-028 study of pembrolizumab (MK-3475). J Clin Oncol.

[29] Kojima T, Hara H, Yamaguchi K, Hironaka S, Iwasa S, Kato K, Tsushima T, Yasui H, Ura T, Muro K (2016). Phase II study of nivolumab (ONO-4538/BMS-936558) in patients with esophageal cancer: Preliminary report of overall survival. J Clin Oncol.

[30] Bass AJ, Vesteinn T, Ilya S, Reynolds SM, Michael M, Brady B, Toshinori H, Laird PW, Christina C, Hui S, Weisenberger DJ, Nikolaus S, Ronglai S, Nils W, Kelsen DP, Reanne B, Andy C, Katayoon K, Mungall AJ, Gordon Robertson A, Payal S, Andrew C, Gad G, Yingchun L, Noble MS, Chandra P, Carrie S, Amaro T-W, Rehan A, Ju-Seog L, Wenbin L, Mills GB, Da Y, Wei Z, Angeliki P, Michael P, Margaret G, Blanca Piazuelo M, Schneider BG, Jihun K, Alex B, Margi S, Demchok JA, Rabkin CS, Willis JE, Sam N, Katherine G, Beer DG, Arjun P, Raphael BJ, Hsin-Ta W, Robert O, Kim HK, Jay B, Leraas KM, Lichtenberg TM, Stephanie W, Michael ML, Maciej W, Ryo S, Gad G, Carrie S, Lawrence MS, Kristian C, Lee L, Sheila F, Gabriel SB, Lander ES, Li D, Beifang N, Adrian A, Miruna B, Inanc B, Reanne B, Denise B, Yaron SN B, Rebecca C, Andy C, Justin C, Eric C, Chun H-JE, Amanda C, Noreen D, Ranabir G, Holt RA, Steven JM J, Katayoon K, Darlene L, Li HA, Emilia L, Yussanne M, Marra MA, Michael M, Moore RA, Mungall AJ, Mungall KL, Ka Ming N, Gordon Robertson A, Schein JE, Payal S, Angela T, Nina T, Rameen B, Carter SL, Cherniack AD, Juok C, Kristian C, Daniel DC, Scott F, Sheila F, Gabriel SB, Nils G, Heiman DI, Joonil J, Jaegil K, Lander ES, Lawrence MS, Lee L, Pei L, Matthew M, Ojesina AI, Chandra Sekhar P, Gordon S, Schumacher SE, Carrie S, Petar S, Barbara T, Amaro T-W, Doug V, Mara R, Zack TI, Hailei Z, Lihua Z, Alexei P, Netty S, Michael P, Semin L, Jianhua Z, Mahadeshwar HS, Jiabin T, Xiaojia R, Sahil S, Lixing Y, Xu AW, Xingzhi S, Angeliki P, Ruibin X, Bristow CA, Angela H, Jonathan S, Lynda C, Park PJ, Raju K, Rehan A, Shiyun L, Wenbin L, Arvind R, Weinstein JN, Sang-Bae K, Ju-Seog L, Yiling L, Gordon M, Laird PW, Toshinori H, Weisenberger DJ, Bootwalla MS, Lai PH, Hui S, Triche T, Van Den Berg DJ, Baylin SB, Herman JG, Gad G, Lynda C, Yingchun L, Murray BA, Noble MS, Arman Askoy RB, Giovanni C, Gideon D, Jianjiong G, Benjamin G, Anders J, William L, Ricardo R, Chris S, Nikolaus S, Yasin S, Rileen S, Onur Sumer S, Yichao S, Nils W, Vésteinn T, Brady B, Lisa I, Kramer RW, Richard K, Michael M, Reynolds SM, Hector R, Natalie T, Ilya S, Sam N, David H, Stuart JM, Rehan A, Shiyun L, Wenbin L, Arvind R, Weinstein JN, Roeland GW V, Mills GB, Mark DM L, Raphael BJ, Hsin-Ta W, Taylor BS, Black AD, Jay B, Julie Ann C, Gastier-Foster JM, Carmen H, Leraas KM, Lichtenberg TM, Cynthia MA, Ramirez NC, Tabler TR, Lisa W, Erik Z, Robert P, Daniel L, Cynthia MA, Ramirez NC, Tabler TR, Lisa W, Erik Z, Robert P, Daniel C, Johanna G, Kevin L, Erin C, David M, Scott M, Joseph P, Troy S, Candace S, Mark S, Christopher B, Jae-Hyuk L, Konstantin F, Georgy M, Olga P, Olga V, Dmitry B, Oleg D, Kimryn Rathmell W, Jakub B, Matthew I, Konstanty K, Witold K, Radoslaw L, Ewa L, Andrzej M, Dawid M, Pawel M, Arkadiusz S, Suchorska WM, Honorata T, Marek T, Maciej W, Raafat A-M, Joseph B, Jennifer B, Mary I, Brenda R, Sun-Young K, Robert P, Johanna G, Ariane K, David M, Scott M, Troy S, Candace S, Erin C, Iakovina A, Jay E, John B, Monique A, Do-Youn P, Rajiv D, James L, Rodney L, Janjigian YY, Kelsen DP, Eunjung C, Marc L, Laura T, McCall SJ, Park YS, Jae-Ho C, Jaffer A, Constanza Camargo M, Shelley A, Brenda A, Jensen MA, Todd P, Rohini R, Jessica W, Yunhu W, Demchok JA, Greg E, Mills Shaw KR, Margi S, Roy T, Zhining W, Liming Y, Jean Claude Z, Tanja D, Hutter CM, Sofia HJ, Robert B, Sudha C, Jia L (2014). Comprehensive molecular characterization of gastric adenocarcinoma. Nature.

[31] Derks S, Liao X, Chiaravalli AM, Xu X, Camargo MC, Solcia E, Sessa F, Fleitas T, Freeman GJ, Rodig SJ (2016). Abundant PD-L1 expression in Epstein-Barr Virus-infected gastric cancers. Oncotarget.

[32] Ma C, Patel K, Singhi AD, Ren B, Zhu B, Shaikh F, Sun W (2016). Programmed Death-Ligand 1 Expression Is Common in Gastric Cancer Associated With Epstein-Barr Virus or Microsatellite Instability. Am J Surg Pathol.

[33] Saito R, Abe H, Kunita A, Yamashita H, Seto Y, Fukayama M (2017). Overexpression and gene amplification of PD-L1 in cancer cells and PD-L1+ immune cells in Epstein-Barr virus-associated gastric cancer: the prognostic implications. Mod Pathol.

[34] Zhang M, Dong Y, Liu H, Wang Y, Zhao S, Xuan Q, Wang Y, Zhang Q (2016). The clinicopathological and prognostic significance of PD-L1 expression in gastric cancer: a meta-analysis of 10 studies with 1,901 patients. Sci Rep.

[35] Muro K, Chung HC, Shankaran V, Geva R, Catenacci D, Gupta S, Eder JP, Golan T, Le DT, Burtness B (2016). Pembrolizumab for patients with PD-L1-positive advanced gastric cancer (KEYNOTE-012): a multicentre, open-label, phase 1b trial. Lancet Oncol.

[36] Kang Y-K, Satoh T, Ryu M-H, Chao Y, Kato K, Chung HC, Chen J-S, Muro K, Kang WK, Yoshikawa T (2017). Nivolumab (ONO-4538/BMS-936558) as salvage treatment after second or later-line chemotherapy for advanced gastric or gastro-esophageal junction cancer (AGC): A double-blinded, randomized, phase III trial. J Clin Oncol.

[37] Arciero CA, Sigurdson ER (2006). Liver-directed therapies for hepatocellular carcinoma. J Natl Compr Canc Netw.

[38] European Association For The Study Of The L, European Organisation For R, Treatment Of C (2012). EASL-EORTC clinical practice guidelines: management of hepatocellular carcinoma. J Hepatol.

[39] Fong ZV, Tanabe KK (2014). The clinical management of hepatocellular carcinoma in the United States, Europe, and Asia: a comprehensive and evidence-based comparison and review. Cancer.

[40] El-Serag HB (2011). Hepatocellular carcinoma. N Engl J Med.

[41] Budhu A, Forgues M, Ye QH, Jia HL, He P, Zanetti KA, Kammula US, Chen Y, Qin LX, Tang ZY, Wang XW (2006). Prediction of venous metastases, recurrence, and prognosis in hepatocellular carcinoma based on a unique immune response signature of the liver microenvironment. Cancer Cell.

[42] Tatsumi T, Takehara T, Katayama K, Mochizuki K, Yamamoto M, Kanto T, Sasaki Y, Kasahara A, Hayashi N (1997). Expression of costimulatory molecules B7-1 (CD80) and B7-2 (CD86) on human hepatocellular carcinoma. Hepatology.

[43] Fujiwara K, Higashi T, Nouso K, Nakatsukasa H, Kobayashi Y, Uemura M, Nakamura S, Sato S, Hanafusa T, Yumoto Y (2004). Decreased expression of B7 costimulatory molecules and major histocompatibility complex class-I in human hepatocellular carcinoma. J Gastroenterol Hepatol.

[44] Matsui M, Machida S, Itani-Yohda T, Akatsuka T (2002). Downregulation of the proteasome subunits, transporter, and antigen presentation in hepatocellular carcinoma, and their restoration by interferon-gamma. J Gastroenterol Hepatol.

[45] Cariani E, Pilli M, Zerbini A, Rota C, Olivani A, Pelosi G, Schianchi C, Soliani P, Campanini N, Silini EM (2012). Immunological and molecular correlates of disease recurrence after liver resection for hepatocellular carcinoma. PLoS One.

[46] Zeng Z, Shi F, Zhou L, Zhang MN, Chen Y, Chang XJ, Lu YY, Bai WL, Qu JH, Wang CP (2011). Upregulation of circulating PD-L1/PD-1 is associated with poor post-cryoablation prognosis in patients with HBV-related hepatocellular carcinoma. PLoS One.

[47] Gao Q, Wang XY, Qiu SJ, Yamato I, Sho M, Nakajima Y, Zhou J, Li BZ, Shi YH, Xiao YS (2009). Overexpression of PD-L1 significantly associates with tumor aggressiveness and postoperative recurrence in human hepatocellular carcinoma. Clin Cancer Res.

[48] Wang BJ, Bao JJ, Wang JZ, Wang Y, Jiang M, Xing MY, Zhang WG, Qi JY, Roggendorf M, Lu MJ, Yang DL (2011). Immunostaining of PD-1/PD-Ls in liver tissues of patients with hepatitis and hepatocellular carcinoma. World J Gastroenterol.

[49] Jung HI, Jeong D, Ji S, Ahn TS, Bae SH, Chin S, Chung JC, Kim HC, Lee MS, Baek MJ (2017). Overexpression of PD-L1 and PD-L2 Is Associated with Poor Prognosis in Patients with Hepatocellular Carcinoma. Cancer Res Treat.

[50] Kuang DM, Zhao Q, Peng C, Xu J, Zhang JP, Wu C, Zheng L (2009). Activated monocytes in peritumoral stroma of hepatocellular carcinoma foster immune privilege and disease progression through PD-L1. J Exp Med.

[51] El-Khoueiry AB, Sangro B, Yau T, Crocenzi TS, Kudo M, Hsu C, Kim TY, Choo SP, Trojan J, Welling THR (2017). Nivolumab in patients with advanced hepatocellular carcinoma (CheckMate 040): an open-label, non-comparative, phase 1/2 dose escalation and expansion trial. Lancet.

[52] Chen ML, Yan BS, Lu WC, Chen MH, Yu SL, Yang PC, Cheng AL (2014). Sorafenib relieves cell-intrinsic and cell-extrinsic inhibitions of effector T cells in tumor microenvironment to augment antitumor immunity. Int J Cancer.

[53] Gani F, Nagarajan N, Kim Y, Zhu Q, Luan L, Bhaijjee F, Anders RA, Pawlik TM (2016). Program Death 1 Immune Checkpoint and Tumor Microenvironment: Implications for Patients With Intrahepatic Cholangiocarcinoma. Ann Surg Oncol.

[54] Ye Y, Zhou L, Xie X, Jiang G, Xie H, Zheng S (2009). Interaction of B7-H1 on intrahepatic cholangiocarcinoma cells with PD-1 on tumor-infiltrating T cells as a mechanism of immune evasion. J Surg Oncol.

[55] Bang YJ, Doi T, De Braud F, Piha-Paul S, Hollebecque A, Abdul Razak AR, Lin CC, Ott PA, He AR, Yuan SS, Koshiji M, Lam B, Aggarwal R (2015). 525 Safety and efficacy of pembrolizumab (MK-3475) in patients (pts) with advanced biliary tract cancer: Interim results of KEYNOTE-028. European Journal of Cancer.

[56] Notta F, Chan-Seng-Yue M, Lemire M, Li Y, Wilson GW, Connor AA, Denroche RE, Liang SB, Brown AM, Kim JC (2016). A renewed model of pancreatic cancer evolution based on genomic rearrangement patterns. Nature.

[57] Erkan M, Hausmann S, Michalski CW, Fingerle AA, Dobritz M, Kleeff J, Friess H (2012). The role of stroma in pancreatic cancer: diagnostic and therapeutic implications. Nat Rev Gastroenterol Hepatol.

[58] Bazhin AV, Shevchenko I, Umansky V, Werner J, Karakhanova S (2014). Two immune faces of pancreatic adenocarcinoma: possible implication for immunotherapy. Cancer Immunol Immunother.

[59] Royal RE, Levy C, Turner K, Mathur A, Hughes M, Kammula US, Sherry RM, Topalian SL, Yang JC, Lowy I, Rosenberg SA (2010). Phase 2 trial of single agent Ipilimumab (anti-CTLA-4) for locally advanced or metastatic pancreatic adenocarcinoma. J Immunother.

[60] Brahmer JR, Tykodi SS, Chow LQ, Hwu WJ, Topalian SL, Hwu P, Drake CG, Camacho LH, Kauh J, Odunsi K (2012). Safety and activity of anti-PD-L1 antibody in patients with advanced cancer. N Engl J Med.

[61] Segal NH, Antonia SJ, Brahmer JR, Maio M, Blake-Haskins A, Li X, Vasselli J, Ibrahim RA, Lutzky J, Khleif S (2014). Preliminary data from a multi-arm expansion study of MEDI4736, an anti-PD-L1 antibody. J Clin Oncol.

[62] Ionov Y, Peinado MA, Malkhosyan S, Shibata D, Perucho M (1993). Ubiquitous somatic mutations in simple repeated sequences reveal a new mechanism for colonic carcinogenesis. Nature.

[63] Thibodeau SN, Bren G, Schaid D (1993). Microsatellite instability in cancer of the proximal colon. Science.

[64] Hewish M, Lord CJ, Martin SA, Cunningham D, Ashworth A (2010). Mismatch repair deficient colorectal cancer in the era of personalized treatment. Nat Rev Clin Oncol.

[65] Eshleman JR, Lang EZ, Bowerfind GK, Parsons R, Vogelstein B, Willson JK, Veigl ML, Sedwick WD, Markowitz SD (1995). Increased mutation rate at the hprt locus accompanies microsatellite instability in colon cancer. Oncogene.

[66] Le DT, Uram JN, Wang H, Bartlett BR, Kemberling H, Eyring AD, Skora AD, Luber BS, Azad NS, Laheru D (2015). PD-1 Blockade in Tumors with Mismatch-Repair Deficiency. N Engl J Med.

[67] Overman MJ, Kopetz S, McDermott RS, Leach J, Lonardi S, Lenz H-J, Morse MA, Desai J, Hill A, Axelson MD, Moss RA, Lin C-S, Goldberg M, Andre T. Nivolumab ± ipilimumab in treatment (tx) of patients (pts) with metastatic colorectal cancer (mCRC) with and without high microsatellite instability (MSI-H): CheckMate-142 interim results. J Clin Oncol. 2016;34(15_suppl):3501–1.

[68] Le DT, Uram JN, Wang H, Bartlett B, Kemberling H, Eyring A, Azad NS, Laheru D, Donehower RC, Crocenzi TS (2016). Programmed death-1 blockade in mismatch repair deficient colorectal cancer. J Clin Oncol.

[69] Overman MJ, Lonardi S, Leone F, McDermott RS, Morse MA, Wong KYM, Neyns B, Leach JL, Alfonso PG, Lee JJ (2017). Nivolumab in patients with DNA mismatch repair deficient/microsatellite instability high metastatic colorectal cancer: Update from CheckMate 142. J Clin Oncol.

[70] Bendell JC, Kim TW, Goh BC, Wallin J, Oh D-Y, Han S-W, Lee CB, Hellmann MD, Desai J, Lewin JH (2016). Clinical activity and safety of cobimetinib (cobi) and atezolizumab in colorectal cancer (CRC). J Clin Oncol.

[71] Ouhoummane N, Steben M, Coutlee F, Vuong T, Forest P, Rodier C, Louchini R, Duarte E, Brassard P (2013). Squamous anal cancer: patient characteristics and HPV type distribution. Cancer Epidemiol.

[72] Palefsky JM, Giuliano AR, Goldstone S, Moreira ED, Aranda C, Jessen H, Hillman R, Ferris D, Coutlee F, Stoler MH (2011). HPV vaccine against anal HPV infection and anal intraepithelial neoplasia. N Engl J Med.

[73] Morris VK, Salem ME, Nimeiri H, Iqbal S, Singh P, Ciombor K, Polite B, Deming D, Chan E, Wade JL (2017). Nivolumab for previously treated unresectable metastatic anal cancer (NCI9673): a multicentre, single-arm, phase 2 study. Lancet Oncol.

[74] Collins FS, Varmus H (2015). A new initiative on precision medicine. N Engl J Med.

[75] Myint ZW, Goel G (2017). Role of modern immunotherapy in gastrointestinal malignancies: a review of current clinical progress. J Hematol Oncol.

[76] Valecha GK, Vennepureddy A, Ibrahim U, Safa F, Samra B, Atallah JP (2017). Anti-PD-1/PD-L1 antibodies in non-small cell lung cancer: the era of immunotherapy. Expert Rev Anticancer Ther.

[77] Bendell JC, Calvo E, Kim JW, Ascierto PA, Sharma P, Ott PA, Bono P, Jaeger D, Evans TRJ, Braud FGD (2016). Safety and activity of nivolumab monotherapy in advanced and metastatic (A/M) gastric or gastroesophageal junction cancer (GC/GEC): Results from the CheckMate-032 study. J Clin Oncol.

[78] Le DT, Bendell JC, Calvo E, Kim JW, Ascierto PA, Sharma P, Ott PA, Bono P, Jaeger D, Evans TRJ (2016). Safety and activity of nivolumab monotherapy in advanced and metastatic (A/M) gastric or gastroesophageal junction cancer (GC/GEC): Results from the CheckMate-032 study. J Clin Oncol.

[79] Liu D, Wang S, Bindeman W (2017). Clinical applications of PD-L1 bioassays for cancer immunotherapy. J Hematol Oncol.

[80] Herbst RS, Baas P, Kim DW, Felip E, Perez-Gracia JL, Han JY, Molina J, Kim JH, Arvis CD, Ahn MJ (2016). Pembrolizumab versus docetaxel for previously treated, PD-L1-positive, advanced non-small-cell lung cancer (KEYNOTE-010): a randomised controlled trial. Lancet.

[81] Jing W, Li M, Zhang Y, Teng F, Han A, Kong L, Zhu H (2016). PD-1/PD-L1 blockades in non-small-cell lung cancer therapy. Onco Targets Ther.

[82] Santarpia M, Giovannetti E, Rolfo C, Karachaliou N, Gonzalez-Cao M, Altavilla G, Rosell R (2016). Recent developments in the use of immunotherapy in non-small cell lung cancer. Expert Rev Respir Med.

[83] Iafolla MAJ, Juergens RA (2017). Update on Programmed Death-1 and Programmed Death-Ligand 1 Inhibition in the Treatment of Advanced or Metastatic Non-Small Cell Lung Cancer. Front Oncol.

[84] Diggs LP, Hsueh EC (2017). Utility of PD-L1 immunohistochemistry assays for predicting PD-1/PD-L1 inhibitor response. Biomark Res.

[85] Yearley JH, Gibson C, Yu N, Moon C, Murphy E, Juco J, Lunceford J, Cheng J, Chow LQM, Seiwert TY (2017). PD-L2 Expression in Human Tumors: Relevance to Anti-PD-1 Therapy in Cancer. Clin Cancer Res.

[86] Bonta I, Isac JF, Meiri E, Bonta D, Rich P (2017). Correlation between tumor mutation burden and response to immunotherapy. J Clin Oncol.

[87] Champiat S, Ferte C, Lebel-Binay S, Eggermont A, Soria JC (2014). Exomics and immunogenics: Bridging mutational load and immune checkpoints efficacy. Oncoimmunology.

[88] Alexandrov LB, Nik-Zainal S, Wedge DC, Aparicio SA, Behjati S, Biankin AV, Bignell GR, Bolli N, Borg A, Borresen-Dale AL (2013). Signatures of mutational processes in human cancer. Nature.

[89] Salipante SJ, Scroggins SM, Hampel HL, Turner EH, Pritchard CC (2014). Microsatellite instability detection by next generation sequencing. Clin Chem.

[90] Lin AY, Lin E (2015). Programmed death 1 blockade, an Achilles heel for MMR-deficient tumors?. J Hematol Oncol.

[91] Le DT, Durham JN, Smith KN, Wang H, Bartlett BR, Aulakh LK, Lu S, Kemberling H, Wilt C, Luber BS, Wong F, Azad NS, Rucki AA, Laheru D, Donehower R, Zaheer A, Fisher GA, Crocenzi TS, Lee JJ, Greten TF, Duffy AG, Ciombor KK, Eyring AD, Lam BH, Joe A, Kang SP, Holdhoff M, Danilova L, Cope L, Meyer C et al: Mismatch-repair deficiency predicts response of solid tumors to PD-1 blockade. Science. 2017. doi:10.1126/science.aan6733. 10.1126/science.aan6733 PMC5576142 28596308

[92] Gubin MM, Artyomov MN, Mardis ER, Schreiber RD (2015). Tumor neoantigens: building a framework for personalized cancer immunotherapy. J Clin Invest.

[93] Van Allen EM, Miao D, Schilling B, Shukla SA, Blank C, Zimmer L, Sucker A, Hillen U, Geukes Foppen MH, Goldinger SM (2015). Genomic correlates of response to CTLA-4 blockade in metastatic melanoma. Science.

[94] Gibney GT, Weiner LM, Atkins MB (2016). Predictive biomarkers for checkpoint inhibitor-based immunotherapy. Lancet Oncol.

[95] Johnson DB, Estrada MV, Salgado R, Sanchez V, Doxie DB, Opalenik SR, Vilgelm AE, Feld E, Johnson AS, Greenplate AR (2016). Melanoma-specific MHC-II expression represents a tumour-autonomous phenotype and predicts response to anti-PD-1/PD-L1 therapy. Nat Commun.

[96] Wang X, Schoenhals JE, Li A, Valdecanas DR, Ye H, Zang F, Tang C, Tang M, Liu CG, Liu X (2017). Suppression of Type I IFN Signaling in Tumors Mediates Resistance to Anti-PD-1 Treatment That Can Be Overcome by Radiotherapy. Cancer Res.

[97] Postow MA, Harding J, Wolchok JD (2012). Targeting immune checkpoints: releasing the restraints on anti-tumor immunity for patients with melanoma. Cancer J.

[98] Harding JJ, El Dika I, Abou-Alfa GK (2016). Immunotherapy in hepatocellular carcinoma: Primed to make a difference?. Cancer.

[99] Charoentong P, Finotello F, Angelova M, Mayer C, Efremova M, Rieder D, Hackl H, Trajanoski Z (2017). Pan-cancer Immunogenomic Analyses Reveal Genotype-Immunophenotype Relationships and Predictors of Response to Checkpoint Blockade. Cell Rep.

[100] Kluger HM, Zito CR, Barr ML, Baine MK, Chiang VL, Sznol M, Rimm DL, Chen L, Jilaveanu LB (2015). Characterization of PD-L1 Expression and Associated T-cell Infiltrates in Metastatic Melanoma Samples from Variable Anatomic Sites. Clin Cancer Res.

[101] Taube JM, Klein A, Brahmer JR, Xu H, Pan X, Kim JH, Chen L, Pardoll DM, Topalian SL, Anders RA (2014). Association of PD-1, PD-1 ligands, and other features of the tumor immune microenvironment with response to anti-PD-1 therapy. Clin Cancer Res.

[102] Formenti SC, Demaria S (2009). Systemic effects of local radiotherapy. Lancet Oncol.

[103] Gabrilovich DI, Ishida T, Nadaf S, Ohm JE, Carbone DP (1999). Antibodies to vascular endothelial growth factor enhance the efficacy of cancer immunotherapy by improving endogenous dendritic cell function. Clin Cancer Res.

[104] Hodi FS, Lawrence D, Lezcano C, Wu X, Zhou J, Sasada T, Zeng W, Giobbie-Hurder A, Atkins MB, Ibrahim N (2014). Bevacizumab plus ipilimumab in patients with metastatic melanoma. Cancer Immunol Res.

[105] Larkin J, Chiarion-Sileni V, Gonzalez R, Grob JJ, Cowey CL, Lao CD, Schadendorf D, Dummer R, Smylie M, Rutkowski P (2015). Combined Nivolumab and Ipilimumab or Monotherapy in Untreated Melanoma. N Engl J Med.

[106] Champiat S, Dercle L, Ammari S, Massard C, Hollebecque A, Postel-Vinay S, Chaput N, Eggermont A, Marabelle A, Soria JC, Ferte C (2017). Hyperprogressive Disease Is a New Pattern of Progression in Cancer Patients Treated by Anti-PD-1/PD-L1. Clin Cancer Res.

[107] Kato S, Goodman A, Walavalkar V, Barkauskas DA, Sharabi A, Kurzrock R. Hyperprogressors after Immunotherapy: Analysis of Genomic Alterations Associated with Accelerated Growth Rate. Clinical cancer research: an official journal of the American Association for Cancer Research. 2017. doi:10.1158/1078-0432.ccr-16-3133. 10.1158/1078-0432.CCR-16-3133 PMC5647162 28351930

[108] Goronzy JJ, Weyand CM (2013). Understanding immunosenescence to improve responses to vaccines. Nat Immunol.

[109] Solana R, Tarazona R, Gayoso I, Lesur O, Dupuis G, Fulop T (2012). Innate immunosenescence: effect of aging on cells and receptors of the innate immune system in humans. Semin Immunol.

[110] Saâda-Bouzid E, Defaucheux C, Karabajakian A, Coloma VP, Servois V, Paoletti X, Even C, Fayette J, Guigay J, Loirat D, Peyrade F, Alt M, Gal J, Le Tourneau C (2017). Hyperprogression during anti-PD-1/PD-L1 therapy in patients with recurrent and/or metastatic head and neck squamous cell carcinoma. Annals of Oncology.

[111] Wade M, Li YC, Wahl GM (2013). MDM2, MDMX and p53 in oncogenesis and cancer therapy. Nat Rev Cancer.

[112] Yan J, Wang ZY, Yang HZ, Liu HZ, Mi S, Lv XX, Fu XM, Yan HM, Zhang XW, Zhan QM, Hu ZW (2011). Timing is critical for an effective anti-metastatic immunotherapy: the decisive role of IFNgamma/STAT1-mediated activation of autophagy. PLoS One.

[113] Reits EA, Hodge JW, Herberts CA, Groothuis TA, Chakraborty M, Wansley EK, Camphausen K, Luiten RM, de Ru AH, Neijssen J (2006). Radiation modulates the peptide repertoire, enhances MHC class I expression, and induces successful antitumor immunotherapy. J Exp Med.

[114] Young KH, Baird JR, Savage T, Cottam B, Friedman D, Bambina S, Messenheimer DJ, Fox B, Newell P, Bahjat KS (2016). Optimizing Timing of Immunotherapy Improves Control of Tumors by Hypofractionated Radiation Therapy. PLoS One.

[115] Zhang B, Bowerman NA, Salama JK, Schmidt H, Spiotto MT, Schietinger A, Yu P, Fu YX, Weichselbaum RR, Rowley DA (2007). Induced sensitization of tumor stroma leads to eradication of established cancer by T cells. J Exp Med.

[116] Deng L, Liang H, Burnette B, Beckett M, Darga T, Weichselbaum RR, Fu YX (2014). Irradiation and anti-PD-L1 treatment synergistically promote antitumor immunity in mice. J Clin Invest.

[117] Seung SK, Curti BD, Crittenden M, Walker E, Coffey T, Siebert JC, Miller W, Payne R, Glenn L, Bageac A, Urba WJ (2012). Phase 1 study of stereotactic body radiotherapy and interleukin-2--tumor and immunological responses. Sci Transl Med.

[118] Pinnamaneni R, Hegde AM, Cherukuri SD, Arastu HH, Bowling M, Leinweber C, Stroud CRG, Cherry CR, Walker PR (2017). Sequence of stereotactic ablative radiotherapy and immune checkpoint blockade in the treatment of metastatic lung cancer. J Clin Oncol.

[119] Hellmann MD, Li BT, Chaft JE, Kris MG (2016). Chemotherapy remains an essential element of personalized care for persons with lung cancers. Ann Oncol.

[120] Lynch TJ, Bondarenko I, Luft A, Serwatowski P, Barlesi F, Chacko R, Sebastian M, Neal J, Lu H, Cuillerot JM, Reck M (2012). Ipilimumab in combination with paclitaxel and carboplatin as first-line treatment in stage IIIB/IV non-small-cell lung cancer: results from a randomized, double-blind, multicenter phase II study. J Clin Oncol.

[121] Vanneman M, Dranoff G (2012). Combining immunotherapy and targeted therapies in cancer treatment. Nat Rev Cancer.

[122] Araki K, Ellebedy AH, Ahmed R (2011). TOR in the immune system. Curr Opin Cell Biol.

[123] Procaccini C, De Rosa V, Galgani M, Abanni L, Cali G, Porcellini A, Carbone F, Fontana S, Horvath TL, La Cava A, Matarese G (2010). An oscillatory switch in mTOR kinase activity sets regulatory T cell responsiveness. Immunity.

[124] Wang Y, Camirand G, Lin Y, Froicu M, Deng S, Shlomchik WD, Lakkis FG, Rothstein DM (2011). Regulatory T cells require mammalian target of rapamycin signaling to maintain both homeostasis and alloantigen-driven proliferation in lymphocyte-replete mice. J Immunol.

[125] Finn OJ (2003). Cancer vaccines: between the idea and the reality. Nat Rev Immunol.

[126] Tabi Z, Man S (2006). Challenges for cancer vaccine development. Adv Drug Deliv Rev.

[127] Srinivasan R, Van Epps DE (2006). Specific active immunotherapy of cancer: potential and perspectives. Rev Recent Clin Trials.

[128] Kudo M (2017). Immune Checkpoint Inhibition in Hepatocellular Carcinoma: Basics and Ongoing Clinical Trials. Oncology.

[129] Yamada Y, Nishina T, Iwasa S, Shitara K, Muro K, Esaki T, Hironaka S, Yamaguchi K, Machida N, Satoh T (2015). A phase I dose expansion trial of avelumab (MSB0010718C), an anti-PD-L1 antibody, in Japanese patients with advanced gastric cancer. J Clin Oncol.

[130] Morris VK, Ciombor KK, Salem ME, Nimeiri HS, Iqbal S, Singh PP, Polite BN, Deming DA, Chan E, Wade JL (2016). NCI9673: A multi-institutional eETCTN phase II study of nivolumab in refractory metastatic squamous cell carcinoma of the anal canal (SCCA). J Clin Oncol.

[131] Wainberg ZA, Segal NH, Jaeger D, Lee K-H, Marshall J, Antonia SJ, Butler M, Sanborn RE, Nemunaitis JJ, Carlson CA (2017). Safety and clinical activity of durvalumab monotherapy in patients with hepatocellular carcinoma (HCC). J Clin Oncol.

[132] Ott PA, Piha-Paul SA, Munster P, Pishvaian MJ, Van Brummelen E, Cohen R, Gomez-Roca C, Ejadi S, Stein M, Chan E, Simonelli M, Morosky A, Yuan SS, Koshiji M, Bennouna J (2015). 500 Pembrolizumab (MK- 3475) for PD-L1-positive squamous cell carcinoma (SCC) of the anal canal: Preliminary safety and efficacy results from KEYNOTE-028. European Journal of Cancer.

[133] Kudo T, Hamamoto Y, Kato K, Ura T, Kojima T, Tsushima T, Hironaka S, Hara H, Satoh T, Iwasa S (2017). Nivolumab treatment for oesophageal squamous-cell carcinoma: an open-label, multicentre, phase 2 trial. Lancet Oncol.

[134] Herbst RS, Gordon MS, Fine GD, Sosman JA, Soria J-C, Hamid O, Powderly JD, Burris HA, Mokatrin A, Kowanetz M (2013). A study of MPDL3280A, an engineered PD-L1 antibody in patients with locally advanced or metastatic tumors. J Clin Oncol.

[135] Kim JH (2016). Comparison of the RECIST 1.0 and RECIST 1.1 in patients treated with targeted agents: a pooled analysis and review. Oncotarget.

[136] Chiou VL, Burotto M (2015). Pseudoprogression and Immune-Related Response in Solid Tumors. J Clin Oncol.

[137] Ades F, Yamaguchi N (2015). WHO, RECIST, and immune-related response criteria: is it time to revisit pembrolizumab results?. Ecancermedicalscience.

[138] Wolchok JD, Hoos A, O’Day S, Weber JS, Hamid O, Lebbe C, Maio M, Binder M, Bohnsack O, Nichol G (2009). Guidelines for the evaluation of immune therapy activity in solid tumors: immune-related response criteria. Clin Cancer Res.

[139] Merlano M, Occelli M, Garrone O (2016). Immune-related response criteria: light and shadows. ESMO Open.

[140] Zaretsky JM, Garcia-Diaz A, Shin DS, Escuin-Ordinas H, Hugo W, Hu-Lieskovan S, Torrejon DY, Abril-Rodriguez G, Sandoval S, Barthly L (2016). Mutations Associated with Acquired Resistance to PD-1 Blockade in Melanoma. N Engl J Med.

[141] Ribas A, Hamid O, Daud A, Hodi FS, Wolchok JD, Kefford R, Joshua AM, Patnaik A, Hwu WJ, Weber JS (2016). Association of Pembrolizumab With Tumor Response and Survival Among Patients With Advanced Melanoma. JAMA.

[142] Koyama S, Akbay EA, Li YY, Herter-Sprie GS, Buczkowski KA, Richards WG, Gandhi L, Redig AJ, Rodig SJ, Asahina H (2016). Adaptive resistance to therapeutic PD-1 blockade is associated with upregulation of alternative immune checkpoints. Nat Commun.

[143] Bernard Escudier, Robert J. Motzer, Padmanee Sharma, John Wagstaff, Elizabeth R. Plimack, Hans J. Hammers, Frede Donskov, Howard Gurney, Jeffrey A. Sosman, Pawel G. Zalewski, Ulrika Harmenberg, David F. McDermott, Toni K. Choueiri, Martin Richardet, Yoshihiko Tomita, Alain Ravaud, Justin Doan, Huanyu Zhao, Helene Hardy, Saby George, (2017) Treatment Beyond Progression in Patients with Advanced Renal Cell Carcinoma Treated with Nivolumab in CheckMate 025. European Urology 10.1016/j.eururo.2017.03.037 28410865

[144] Gandara DR, Pawel JV, Sullivan RN, Helland A, Han J-Y, Aix SP, Rittmeyer A, Barlesi F, Kubo T, Park K (2017). Impact of atezolizumab (atezo) treatment beyond disease progression (TBP) in advanced NSCLC: Results from the randomized phase III OAK study. J Clin Oncol.

[145] Leger PD, Rothschild S, Castellanos E, Pillai RN, York SJ, Horn L (2017). Response to salvage chemotherapy following exposure to immune checkpoint inhibitors in patients with non-small cell lung cancer. J Clin Oncol.

[146] Rothschild SI, Leger P, Castellanos EL, Pillai RN, York SJ, Horn L (2017). 91PD_PRResponse to salvage chemotherapy following exposure to PD-1/PD-L1 inhibitors in patients with NSCLC. Ann Oncol.

[147] Tartari F, Santoni M, Burattini L, Mazzanti P, Onofri A, Berardi R (2016). Economic sustainability of anti-PD-1 agents nivolumab and pembrolizumab in cancer patients: Recent insights and future challenges. Cancer Treat Rev.

[148] Neumann PJ, Cohen JT, Weinstein MC (2014). Updating cost-effectiveness--the curious resilience of the $50,000-per-QALY threshold. N Engl J Med.

[149] Xiao Min W, Liu Bao P, Jin An M, Yuan Jian L (2017). Economic evaluation of nivolumab as a secondline treatment for advanced renal cell carcinoma from US and Chinese perspectives. Cancer.

[150] Matter-Walstra K, Schwenkglenks M, Aebi S, Dedes K, Diebold J, Pietrini M, Klingbiel D, von Moos R, Gautschi O (2016). A Cost-Effectiveness Analysis of Nivolumab versus Docetaxel for Advanced Nonsquamous NSCLC Including PD-L1 Testing. J Thorac Oncol.

[151] Doi T, Piha-Paul SA, Jalal SI, Mai-Dang H, Yuan S, Koshiji M, Csiki I, Bennouna J (2015). Pembrolizumab (MK-3475) for patients (pts) with advanced esophageal carcinoma: Preliminary results from KEYNOTE-028. J Clin Oncol.

[152] Chung HC, Arkenau H-T, Wyrwicz L, Oh D-Y, Lee K-W, Infante JR, Chin KM, Heydebreck AV, Kang Y-K, Safran H (2016). Safety, PD-L1 expression, and clinical activity of avelumab (MSB0010718C), an anti-PD-L1 antibody, in patients with advanced gastric or gastroesophageal junction cancer. J Clin Oncol.

[153] Fuchs CS, Doi T, Jang RW-J, Muro K, Satoh T, Machado M, Sun W, Jalal SI, Shah MA, Metges J-P (2017). KEYNOTE-059 cohort 1: Efficacy and safety of pembrolizumab (pembro) monotherapy in patients with previously treated advanced gastric cancer. J Clin Oncol.

[154] Bang Y-J, Muro K, Fuchs CS, Golan T, Geva R, Hara H, Jalal SI, Borg C, Doi T, Wainberg ZA (2017). KEYNOTE-059 cohort 2: Safety and efficacy of pembrolizumab (pembro) plus 5-fluorouracil (5-FU) and cisplatin for first-line (1 L) treatment of advanced gastric cancer. J Clin Oncol.

[155] Janjigian YY, Bendell JC, Calvo E, Kim JW, Ascierto PA, Sharma P, Ott PA, Bono P, Jaeger D, Evans TRJ (2016). CheckMate-032: Phase I/II, open-label study of safety and activity of nivolumab (nivo) alone or with ipilimumab (ipi) in advanced and metastatic (A/M) gastric cancer (GC). J Clin Oncol.

[156] Bang Y-J, Chung H-C, Shankaran V, Geva R, Catenacci DVT, Gupta S, Eder JP, Berger R, Gonzalez EJ, Ray A (2015). Relationship between PD-L1 expression and clinical outcomes in patients with advanced gastric cancer treated with the anti-PD-1 monoclonal antibody pembrolizumab (MK-3475) in KEYNOTE-012. J Clin Oncol.

[157] Crocenzi TS, El-Khoueiry AB, Yau TC, Melero I, Sangro B, Kudo M, Hsu C, Trojan J, Kim T-Y, Choo S-P (2017). Nivolumab (nivo) in sorafenib (sor)-naive and -experienced pts with advanced hepatocellular carcinoma (HCC): CheckMate 040 study. J Clin Oncol.

[158] Melero I, Sangro B, Yau TC, Hsu C, Kudo M, Crocenzi TS, Kim T-Y, Choo S, Trojan J, Meyer T (2017). Nivolumab dose escalation and expansion in patients with advanced hepatocellular carcinoma (HCC): The CheckMate 040 study. J Clin Oncol.

[159] El-Khoueiry AB, Sangro B, Yau TC, Crocenzi TS, Welling TH, Yeo W, Chopra A, Anderson J, Cruz CMD, Lang L (2016). Phase I/II safety and antitumor activity of nivolumab (nivo) in patients (pts) with advanced hepatocellular carcinoma (HCC): Interim analysis of the CheckMate-040 dose escalation study. J Clin Oncol.

[160] El-Khoueiry AB, Melero I, Crocenzi TS, Welling TH, Yau TC, Yeo W, Chopra A, Grosso J, Lang L, Anderson J (2015). Phase I/II safety and antitumor activity of nivolumab in patients with advanced hepatocellular carcinoma (HCC): CA209-040. J Clin Oncol.

[161] Shahda S, Noonan AM, Bekaii-Saab TS, O’Neil BH, Sehdev A, Shaib WL, Helft PR, Loehrer PJ, Tong Y, Liu Z, El-Rayes BF (2017). A phase II study of pembrolizumab in combination with mFOLFOX6 for patients with advanced colorectal cancer. J Clin Oncol.

[162] Leal AD, Paludo J, Finnes HD, Grothey A (2017). Response to pembrolizumab in patients with mismatch repair deficient (dMMR) colorectal cancer (CRC). J Clin Oncol.

[163] Andre T, Lonardi S, Wong KYM, Morse M, McDermott RS, Hill AG, Hendlisz A, Lenz H-J, Leach JW, Moss RA (2017). Combination of nivolumab (nivo) + ipilimumab (ipi) in the treatment of patients (pts) with deficient DNA mismatch repair (dMMR)/high microsatellite instability (MSI-H) metastatic colorectal cancer (mCRC): CheckMate 142 study. J Clin Oncol.

[164] Segal NH, Kemeny NE, Cercek A, Reidy DL, Raasch PJ, Warren P, Hrabovsky AE, Campbell N, Shia J, Goodman KA (2016). Non-randomized phase II study to assess the efficacy of pembrolizumab (Pem) plus radiotherapy (RT) or ablation in mismatch repair proficient (pMMR) metastatic colorectal cancer (mCRC) patients. J Clin Oncol.

[165] Le DT, Uram JN, Wang H, Bartlett B, Kemberling H, Eyring A, Skora A, Azad NS, Laheru DA, Donehower RC (2015). PD-1 blockade in tumors with mismatch repair deficiency. J Clin Oncol.

[166] Ott PA, Piha-Paul SA, Munster P, Pishvaian MJ, van Brummelen EMJ, Cohen RB, Gomez-Roca C, Ejadi S, Stein M, Chan E (2017). Safety and antitumor activity of the anti-PD-1 antibody pembrolizumab in patients with recurrent carcinoma of the anal canal. Ann Oncol.

